# A Prefrontal-Hippocampal Comparator for Goal-Directed Behavior: The Intentional Self and Episodic Memory

**DOI:** 10.3389/fnbeh.2015.00323

**Published:** 2015-11-25

**Authors:** Robert Numan

**Affiliations:** Psychology Department, Santa Clara UniversitySanta Clara, CA, USA

**Keywords:** prefrontal cortex, hippocampus, medial septum, episodic memory, comparator, volition, goal-directed behavior, theta rhythm

## Abstract

The hypothesis of this article is that the interactions between the prefrontal cortex and the hippocampus play a critical role in the modulation of goal-directed self-action and the strengthening of episodic memories. We describe various theories that model a comparator function for the hippocampus, and then elaborate the empirical evidence that supports these theories. One theory which describes a prefrontal-hippocampal comparator for voluntary action is emphasized. Action plans are essential for successful goal-directed behavior, and are elaborated by the prefrontal cortex. When an action plan is initiated, the prefrontal cortex transmits an efference copy (or corollary discharge) to the hippocampus where it is stored as a working memory for the action plan (which includes the expected outcomes of the action plan). The hippocampus then serves as a response intention-response outcome working memory comparator. Hippocampal comparator function is enabled by the hippocampal theta rhythm allowing the hippocampus to compare expected action outcomes to actual action outcomes. If the expected and actual outcomes match, the hippocampus transmits a signal to prefrontal cortex which strengthens or consolidates the action plan. If a mismatch occurs, the hippocampus transmits an error signal to the prefrontal cortex which facilitates a reformulation of the action plan, fostering behavioral flexibility and memory updating. The corollary discharge provides the self-referential component to the episodic memory, affording the personal and subjective experience of what behavior was carried out, when it was carried out, and in what context (where) it occurred. Such a perspective can be applied to episodic memory in humans, and episodic-like memory in non-human animal species.

## Introduction

In its simplest form, a comparator is a device for making comparisons (usually comparing something against a standard measure). A common use of this term in behavioral neuroscience involves neural mechanisms used to compare the expected outcomes of behavior with the actual outcomes of behavior (Numan, 1978, 2000). Neuronal comparators are often described as match/mismatch detectors since they determine if actual behavioral outcomes agree (match) or disagree (mismatch) with the expected outcomes of a behavioral action (Duncan et al., 2012). For example, Von Holst (1954) proposed that the initiation of movement (efference) produced an efference copy (corollary discharge) in the CNS. This efference copy, therefore, was a working-memory of the planned movement and it included the expected outcomes of the planned movement (Clearly, planning is key here, as plans have a prospective focus on outcome expectations). According to Von Holst, the actual outcomes of a planned action (reafference) could now be compared against the expected outcomes encoded in the efference copy, and planning would be modified in case of a mismatch.

Miller et al. (1960) employed a comparator to explain how organisms utilize an action plan to navigate cognitive maps. Their comparator model was proposed to bridge the “theoretical vacuum between cognition and action.” In this model, the comparison process is called a “test” and the comparator was a component of a feedback loop called the “Test-Operate-Test-Exit” (TOTE) unit. If the test determines that the expected and actual behavioral outcomes match, the behavior is terminated (exit). If a mismatch occurs, behavior is continued until a match is achieved. Thus, in their view, the operation of the TOTE unit involves the execution (operate mode) and testing of behavioral plans using working-memory. Based on the experimental data available at that time, they hypothesized that the working-memory for planned actions is formed in the prefrontal cortex, and that the comparator testing occurs in the limbic system.

Anokhin (1969) developed a model of behavioral planning and testing similar to those developed by Von Holst (1954) and Miller et al. (1960). He emphasized the critical importance of the comparator system for the formulation of future behavior; a mismatch between expected and actual outcomes of behavior would result in the formulation of a new program of action.

## Early theories of a hippocampal comparator

To my knowledge, Vinogradova (1970) was the first to formulate a comprehensive description of a comparator function for the hippocampus. She made unit recordings from hippocampal area CA3 in rabbits. Approximately 60% of the neurons showed inhibitory (I) responses to sensory stimuli (e.g., 500 Hz tone), while 40% showed excitatory (E) responses. Following repeated stimulus presentations these responses habituated (returned to spontaneous activity levels). However, slight changes in the stimulus (e.g., pitch change, stimulus duration change, etc.) resulted in a resurgence of responding (E or I) in the habituated neurons. For Vinogradova, these hippocampal neurons appeared to be detecting “the absence of congruency between trace system and actual stimulus.” Most of these neurons (~85%) were multimodal, responding in the manner described to various presented stimuli (tones, flashing lights, clicks, etc.).

Vinogradova (1970) suggested that the hippocampal neurons that responded to a new stimulus with excitation were “novelty detectors,” while those that responded with inhibition were “identity detectors.” The identity detectors are active when sensory information matches sensory expectation (thus, a novel stimulus inhibits the activity of these cells). This activity was thought to inhibit the registration of this stimulus as new. However, under mismatch conditions, these identity detectors were inhibited, allowing the registration (memory formation) of the new stimulus.

Vinogradova remained a staunch supporter of the hippocampal comparator hypothesis, and updated her ideas many times (e.g., 1975) including a final paper (2001) accepted for publication in *Hippocampus* on June 1, 2001, 1 week prior to her death (June 8, 2001). In that 2001 paper, she elaborated her ideas, specifying CA3 as the location of the comparator which compares sensory signals derived from two inputs: The cortex (via perforant path>dentate gyrus>CA3), and the reticular formation (via the medial septum). She also speculated that the pacing of the hippocampal theta rhythm by the medial septum would synchronize these two inputs, facilitating the comparison process.

At about the same time that Vinogradova (1970, 1975) developed her theory of a hippocampal comparator for the detection of sensory novelty, Numan (1972, 1978, 2000) developed his theory of a hippocampal response intention-response outcome working-memory comparator. Numan's theory was influenced by the aforementioned work of Von Holst (1954), Miller et al. (1960) and Anokhin (1969), as well as Vanderwolf's (1969, 1971) discovery of a correlation between the hippocampal theta rhythm and voluntary movement in rats. Like Miller et al. (1960), Numan (1972, 1978) focused on the utility of an action plan (which he called a motor program) when an organism interacted with its environment, and a mechanism for evaluating the effectiveness of the motor program based on feedback derived from the consequences of action. The motor program was described as a molar concept; it did not specify specific movements *per se*, but rather a general action plan for achieving a goal. Numan (1978) hypothesized that the dorsolateral prefrontal cortex (dlPFC; or its homolog in non-primate species), medial septum-diagonal band (MSDB), and dorsal hippocampus (posterior hippocampus in primates) were the core areas of this system, in part, because of the similar difficulties with response regulation (e.g., loss of behavioral flexibility) following damage to these structures (see (Numan, 1978, 2000) for reviews). His theory postulated that the motor program (action plan) was formulated by the dlPFC based on the current environmental context, the motivational and emotional state of the organism, and previous experiential factors (stored memories). Once the motor program was formulated, the dlPFC transmitted an efference copy (corollary discharge) of the program to the hippocampus where it was temporarily stored as a working-memory. This motor program included both an action plan and the expected consequences of action. When the motor program is initiated, the MSDB activates the hippocampal theta rhythm, enabling the hippocampal comparator, allowing reafference (response dependent sensory changes) transmitted to the hippocampus to be compared against the efference copy.

Hence, in the hippocampus, the intended (expected) response outcomes (encoded in the efference copy) are compared with the actual response outcomes derived from reafference. If the expected and actual outcomes match, the hippocampus transmits a signal to dlPFC to strengthen (consolidate) the current action plan. In the case of a mismatch, an error signal is transmitted to dlPFC and a new motor program is formulated based on the consequences of the just completed action. This analysis suggests, therefore, that both the dlPFC and the hippocampus store a working memory (WM) for the action plan; the hippocampal WM serving as a comparator which functions to signal match or mismatch information to strengthen or modify, respectively, the WM for the action plan stored in dlPFC.

In his initial formulation of this theory, Numan (1972) placed the comparator in hippocampal area CA1. He hypothesized that the efference copy was initially stored in hippocampal area CA3. Then, upon response initiation and theta activation, the efference copy was transferred to the hippocampal comparator in CA1.

In 1982, Gray published a monograph on the septohippocampal system which presented a comparator model similar to that described by Numan (1978). Gray located his comparator in the subiculum. Here, information about the current sensory environment, and intended movements in that environment were derived from sensory association cortex and prefrontal cortex, respectively. This information is transmitted to the subiculum via the entorhinal cortex. Predictions about expected outcomes of behavioral actions are also transmitted to the subiculum from the thalamus, and cingulate, temporal and prefrontal cortexes. Thus, this set of inputs provides the comparator with sensory information about the current environment, current and intended motor programs, and the expected outcomes of behavior. Once the behavioral act is initiated, the actual environmental outcomes are transmitted, via entorhinal cortex, to the subiculum for comparison with the expected outcomes. If there is a mismatch between these inputs, an error signal is sent to higher level systems (presumably in prefrontal cortex) that plan and execute motor programs to stop or inhibit the current action program. In the case of a match, the current motor program is maintained.

In both Numan's (1978) and Gray's (1982) models, the inability to detect a mismatch (due to comparator dysfunction) between expected and actual outcomes of behavior (i.e., error detection) would lead to the maintenance of the current action plan. This, of course, would result in an increased probability of response perseveration, which is so common following septohippocampal damage in a variety of species (Numan, 1978; Gray, 1982).

However, it is well known that perseveration following septohippocampal damage, does not typically occur when salient environmental cues (exafference) are available to guide behavior (see Numan, 1978, 2000). This underscores the importance of the septohippocampal system for solving spatial problems (O'Keefe and Nadel, 1978). However, a failure of error detection leading to response perseveration following septohippocampal damage also occurs in non-spatial tasks when salient external cues are not available to guide behavior (e.g., DRL, Go/No-go; see Numan, 1978, 2000; Numan and Klis, 1992; Numan et al., 1995). Numan (1978) suggested that damage to the septohippocampal system impairs behavioral flexibility, in both spatial and non-spatial tasks, when salient external cues are not available to guide behavior. When such salient external cues are available, other brain regions can successfully detect errors and maintain behavioral flexibility when the septohippocampal system is compromised; behavior being regulated by environmental cues that predict positive outcomes (Griffiths et al., 2014). While not universal (e.g., see M'Harzi and Jarrard, 1992), numerous experiments have reported that damage to the septohippocampal system (MSDB, fornix, hippocampus) did not impair, and sometimes facilitated, performance on tasks guided by salient exteroceptive cues (Zola and Mahut, 1973; Aggleton et al., 1986; Eichenbaum et al., 1988; Packard et al., 1989; Ennaceur and Meliani, 1992; Kelsey and Vargas, 1993; Cho and Kesner, 1995; Bussey et al., 1998; Gaffan et al., 2001; Janisewicz and Baxter, 2003).

In this regard, McDonald and White (1995) have suggested that, for rats, spatial discrimination in a maze is dependent on an intact hippocampus only if the cues identifying locations are ambiguous and the rat is allowed to voluntarily move through the maze. Hudon et al. (2003) tested fornix and sham lesioned rats on a radial maze with either intramaze or extramaze cues that were either fixed or variable. Fornix lesions impaired performance only when variable extramaze cues were used. In this case, the cues identifying locations were ambiguous, and control rats could achieve efficient performance by remembering their movement trajectory through the maze. However, the failure of a working memory comparator for voluntary action would disrupt such a strategy and impair the performance of the fornix lesioned rats. As a corollary, Gaffan et al. (2003) found that rats with fornix transection were just as efficient as controls in acquiring incidental learning about allocentric spatial cues when navigation was not required.

Gray (1982) suggests that the septohippocampal system is not involved in working memory. Here, he focuses on the perseverative impairments observed on operant DRL (Differential Reinforcement of Low Rates of Responding) tasks following septohippocampal damage, which he suggests cannot be mediated by working memory. He states that “what changes from moment to moment in a DRL task is not what the animal must remember, but the requirement to respond or not to respond.” However, a failure of response inhibition is not a viable explanation for DRL perseverative responding following septohippocampal lesions in rats (Ellen and Butter, 1969) and cats (Numan and Lubar, 1974) because the lesioned animals show normal response inhibition on this task when an external cue signals the end of the DRL interval. In contrast, a failure of a working memory comparator for voluntary action would prevent the detection and correction of premature DRL responding, leading to perseveration in the absence of a cue signaling the end of the DRL interval. But, in the presence of such a cue, the animal can simply sit and wait for the cue to elicit a stimulus-response procedural habit (Griffiths et al., 2014; see also Young and McNaughton, 2000).

In support of this WM idea, Numan et al. (2004) found that rats with MSDB lesions could normally discriminate immediate contingent (response dependent) reinforcement from non-contingent (response independent) reinforcement, and decreased lever pressing for contingent reinforcements, just like control rats, when contingencies were shifted from positive toward zero (equal probability of reinforcement for responding or not responding). However, such discrimination failed when contingent reinforcements were delayed by 5-s. The lesion effect appeared to be due to a failure for the working memory for voluntary action, which impaired sensitivity to operant contingencies when there was a delay between action and outcome. This failure of volitional memory appears to be mediated by MSDB modulation of hippocampal circuits, as similar results have recently been reported in human patients with damage to the medial temporal lobe (Foerde et al., 2013).

Moreover, Crystal et al. (2013) trained rats, in a radial maze, to discriminate chocolate reward locations obtained by experimenter placement of the rat at the food area vs. reward location obtained by the rat voluntarily walking to the food area. All rats learned this “source memory” by discriminating food areas based on passive placement vs. active movement. Once this task was well learned, temporary inactivation of hippocampal area CA3 with lidocaine eliminated this “source memory”; rats could no longer discriminate food locations based on passive vs. active movement.

A series of experiments by Voss et al. (2011) found that normal human subjects showed better memory for both object recognition and object location when they could actively control (volitional control) their viewing of the objects compared to when they passively viewed the objects. Importantly, predetermined movements to view the objects, lacking volitional control, did not improve memory. Subsequently, human subjects with hippocampal damage failed to show the memory benefit derived from volitional control. Finally, fMRI analysis in normal human subjects found that volitional control was associated with enhanced coordination of activity between the hippocampus and the prefrontal cortex (both dorsolateral and medial prefrontal areas).

These findings suggest that an essential component of episodic memory is the memory for the act of personal doing; not only the memory for past doing (retrospective memory), but also the planning of future doing (prospective memory; also see Gaesser et al., 2013 for the role of human hippocampus and prefrontal cortex in future planning). Such memory for self-referential actions and plans places the self as a core component of episodic memory.

What is the result of comparator failure? First, the match function for correctly completed acts is unavailable and the action plan cannot be strengthened, causing a failure to recall successful task completion (e.g., did I take my pills this morning?). Second, mismatch failure prevents an error signal for incorrect actions, increasing the probability of perseveration. Under these conditions behavior will come under the control of procedural habits and/or semantic memory networks (Zola-Morgan and Squire, 1993; Mishkin et al., 1997; Tulving and Markowitsch, 1998).

Most models of episodic memory focus on the integration of cortically derived spatial, non-spatial, and temporal information by the hippocampus (relational memory). Manns and Eichenbaum (2006) present an excellent review of such neural models of episodic memory. What is lacking from these models, however, is the self-referential component of episodic memory. Perhaps the interaction between the prefrontal cortex and the septohippocampal system provides this self-referential core to episodic memory. In Numan's (1972, 1978, 2000) comparator model, the corollary discharge from prefrontal cortex, stored as a working memory efference copy in the hippocampus provides such a self-referential component, “giving a sense of ‘self’ during behavior” (Poulet and Hedwig, 2007). Such an idea is supported by the work of Kelley et al. (2002) using fMRI in human participants. They found that self-referential judgements selectively engaged the medial prefrontal cortex and improved memory for those judgements compared to other-referential judgements. Moreover, Philippi et al. (2012) found that lesions of the medial prefrontal cortex abolished this self-referential effect on memory.

Klein (2013) also proposes that episodic memory involves not only a sense of time and place but also autonoetic awareness: a self-knowing sense of memory ownership and that episodic memory involves an interaction between the prefrontal cortex and the medial temporal lobe (see also Wheeler et al., 1997; Tulving, 2002; Buckner and Carroll, 2007; Section Episodic Memory of the current manuscript).

## Recent evidence for a hippocampal comparator

Fyhn et al. (2002) provide evidence for a mismatch detector in hippocampal area CA1 of rats. They recorded unit activity from CA1 pyramidal cells while rats learned to find an escape platform in an annular water maze. Once learning was established, CA1 cells were relatively silent. Subsequently, movement of the escape platform to a new location led to an increased firing rate of the CA1 cells when the rat found the location of the moved platform. They suggest that this increased activity is caused by the difference in the new goal location compared to the stored memory of the initially learned location. In contrast, CA1 cells did not increase their firing rates when the platform location was changed on every test trial, suggesting that novelty alone was not responsible for the rate change. Thus, only novelty that contrasted with previously acquired experience lead to an increased firing rate of CA1 cells.

Lisman and Grace (2005) also provide evidence for a comparator in dorsal hippocampal area CA1 of rats. Their comparator detects novelty by comparing expected events, derived from prior memories and relayed to CA1 from CA3, with actual current events (current reality) relayed to CA1 from the cerebral cortex.

A more recent experiment in rats (Lever et al., 2010) also supports a comparator process for hippocampal area CA1 (as suggested above), rather than in the subiculum as suggested by Gray (1982) and McNaughton (2006). Lever et al. found that the firing of CA1 principal cells shifts to a later phase of theta when the rat explores a novel environment. This effect was not observed from cells recorded from the subiculum. They suggest that this CA1 firing theta shift correlates with plasticity in the CA1 place cell representation, and may underlie the neural mechanism required for detecting and encoding novelty (the comparator is probably active in a completely novel environment, because expectations about that environment have not yet been encoded; see our discussion in Section Discussion).

In a series of experiments, using fMRI in human participants, Kumaran and Maguire (2006, 2007a,b, 2009) also provide evidence for associative match-mismatch processes in the hippocampus. They suggest that the hippocampus detects novelty by comparing current sensory input to stored memories; novelty is detected when current sensory input does not match with expectations derived from experience. In their 2006 study, participants first viewed a sequence of four objects (pictures of animals, cars, household objects, etc.) in a specific sequence (A>B>C>D). Following this experiential exposure the participants viewed the same quartet of objects in one of the following sequences: same (A>B>C>D), entirely new (C>A>D>B), or partial novelty where the last two objects in the sequence were reversed (A>B>D>C). They found that left hippocampal activation was greatest in the case of partial novelty. In contrast, hippocampal activation to the entirely new sequence did not differ from activation produced by the same sequence. They, therefore, conclude (as did the Fyhn et al., 2002 study in rats cited above) that the hippocampus does not respond to novelty *per se*, but rather to an associative match-mismatch process. In the partial novelty condition, the first two objects (A>B) activate the expectation, based on the representation of initial exposure, that C>D should follow (pattern completion). When this expectation is violated by the partial mismatch (D>C), the hippocampus becomes activated and generates a mismatch signal. In contrast, they found that the right perirhinal/entorhinal cortex responds to novelty *per se*, showing equally enhanced activation to both the entirely new sequence, and the partial novelty sequence.

Duncan et al. (2009) used fMRI in human participants engaged in a two-object working memory task. They found activation in the posterior hippocampus for both matches and mismatches, and they suggest that the hippocampus maintains a representation of the goal during the delay period for subsequent comparison with the probe. In a subsequent study, Duncan et al. (2012) employed high resolution fMRI in human participants to determine the hippocampal subfield responsible for match/mismatch detection. They found that only hippocampal field CA1 was involved in match/mismatch processes. Interestingly, they found that some voxels in CA1 increased activity to mismatches, while others showed maximal activity to perfect matches and decreased activity when a mismatch occurred. Similar results have been reported by Chen et al. (2011). These findings are compatible with Vinogradova's (1970) novelty and identity detectors, described earlier.

Zou et al. (2009) present data from rats suggesting that hippocampal theta waves (local field potential, 6–9 Hz) reflect the activity of a hippocampal comparator during navigation. Here, a mismatch between expected and actual sensory input was produced by placing rats on a treadmill that was attached to a motor stage that moved along a track. While the rats ran in a forward direction on the treadmill, the rats were moved backward on the track by translocation. Theta power increased only in this condition, not showing increases with forward translocation nor backward translocation *without* locomotion. Aitake et al. (2011) reported similar findings. In both of these cases, theta power increased when there was a mismatch between forward locomotion and backward idiothetic feedback.

These data suggest that the hippocampus serves as an associative match-mismatch comparator under a variety of conditions. However, perhaps the hippocampal comparator is always “online” during these various test conditions, but is not functionally essential for all of them. For example, Ferbinteanu et al. (2011) tested rats in a + maze on a spatial task and a cue task. Excitotoxic hippocampal lesions impaired performance on the spatial task, but not the cue task. In another group of rats, these authors recorded unit responses from dorsal hippocampal CA1 cells while the rats performed either the spatial task or the cue task. They found that journey-dependent activity of the cells occurred during both the spatial task and the cue task, and that such journey-dependent activity was disrupted on error trials in both conditions. Hence, the hippocampus was “online” during both spatial and cue conditions, but was only functionally relevant for performance in the spatial task (as hippocampal damage only impaired performance for the spatial condition). These data are intriguing, and lend support to our view that the hippocampus serves as a response intention-response outcome working memory comparator. Dorsal hippocampal neurons assess response intention-response outcome comparisons under both spatial and the cue conditions. Such hippocampal assessment, however, is only functionally relevant to the spatial task as extra-hippocampal regions can mediate accurate stimulus-response performance under discrete cue conditions via procedural/reference memory systems (Numan, 2000).

## Fronto-hippocampal relations

Numan's comparator theory (1978) states that the dorsolateral prefrontal cortex formulates the action plan and transmits an efference copy (corollary discharge) to the hippocampus. The hippocampus holds this efference copy in working memory to compare its encoded expected outcomes of behavior with actual outcomes. When the hippocampal theta rhythm enables the comparator, the match or mismatch outcomes were signaled back to the prefrontal cortex where the action plan was either strengthened (correct match signal) or modified (error mismatch signal).

Central to this conceptualization, is the two-way communication between the prefrontal cortex and the hippocampus, as well as the theta rhythm enabling the comparator function. There does not appear to be a direct monosynaptic projection from the prefrontal cortex to the hippocampus. However, like other regions of association cortex, prefrontal projections can reach the hippocampus via the entorhinal cortex (Van Hoesen et al., 1972), and perhaps via other anatomical routes that may include the retrosplenial cortex, and the nucleus reuniens of the thalamus (Morris et al., 1999; Vertes, 2006; Vann et al., 2009; Aggleton, 2012; Prasad and Chudasama, 2013; Miller et al., 2014; Griffin, 2015). In contrast, it is now well known that the hippocampus has a monosynaptic glutamatergic projection to prefrontal cortex. In the rat, this monosynaptic path originates in the ventral hippocampus CA1/subiculum and terminates in the medial (prelimbic and medial orbital) prefrontal cortex (Jay and Witter, 1991; Thierry et al., 2000).

This projection to PFC from ventral hippocampus, given the current perspective, is potentially problematic. Numan (1978) emphasized the role of the dorsal hippocampus in his model, and additional reports support a dissociation of function along the dorsoventral axis of the hippocampus, with the dorsal region (posterior hippocampus in humans) mediating cognitive, spatial, and working memory functions while the ventral region (anterior hippocampus in humans) mediates affective behavior (Numan, 1978; Moser and Moser, 1998; Bannerman et al., 1999, 2003; Fanselow and Dong, 2010). In this regard, O'Neill et al. (2013) studying mice on a spatial working memory task in a T-maze, found that the medial prefrontal cortex and the dorsal hippocampus became synchronized at theta frequency, and that the degree of synchrony positively correlated with behavioral performance. They suggest that the synchrony between the dorsal hippocampus and medial prefrontal cortex may be mediated by the ventral hippocampus, which is connected to the dorsal hippocampus and projects directly to the medial prefrontal cortex. In support, they found that if the influence of the ventral hippocampus was computationally or experimentally removed, the synchrony between dorsal hippocampus and medial prefrontal cortex was reduced. This important role of the ventral hippocampus, which mediates affective states, makes sense if we assume that personal self-referential planning contains an affective component.

Functional connectivity analysis has also found connectivity between the dorsolateral prefrontal cortex and hippocampus during navigation in humans (Brown et al., 2014), and connectivity between the dorsomedial prefrontal cortex and dorsal hippocampus in rats navigating a radial maze (Goto and Grace, 2008). Benetti et al. (2009) found impaired performance on a delayed match to sample task in human schizophrenics. This impairment was associated with a decreased functional connectivity between the right posterior hippocampus and the right inferior frontal gyrus.

## Place cells and movement

If the hippocampus serves as a response intention-response outcome working memory comparator, then hippocampal neurons should evidence response-related correlates. There is now abundant evidence to support such a proposal.

The discovery of place cells in the dorsal hippocampal CA1 region of freely moving rats by O'Keefe and Dostrovsky (1971) led to the seminal work of O'Keefe and Nadel (1978) emphasizing the spatial mapping properties of the hippocampus, based primarily on allocentric visual cues. However, non-visual cues can also regulate place cell firing (Sharp et al., 1995), and Save et al. (1998) found that place cell firing in early blind rats was very similar to that recorded from sighted rats. Many investigators, therefore, have suggested that information derived from self-motion (including proprioceptive and vestibular cues, reafferent cues, and corollary discharges) may play an important role in both hippocampal place cell firing and navigation (Gothard et al., 1996; Granger et al., 1996; McNaughton et al., 1996). The control of navigation by self-motion cues is referred to as path integration (Whishaw, 2000). In this regard, Foster et al. (1989) recorded firing rates from hippocampal place cells while rats were allowed to move freely, or when movement was restrained. They found excellent place specificity for these cells when the rats were free to move, but there was an almost complete suppression of place specificity during restraint.

Wiener and Korshunov (1995) recorded unit responses from dorsal CA1 place cells while rats searched for rewards in a square arena. They found that cell firing best correlated with the execution of task behaviors rather than specific spatial locations.

Hetherington and Shapiro (1993) also suggested that movement through a spatial location might be crucial for the maintenance of hippocampal place cell firing. Muller and Kubie (1989) presented evidence that hippocampal place cells may regulate the selection of movement trajectories because their firing pattern correlates best with the rat's subsequent position in space; the spikes appeared to predict where the rat would go. Markus et al. (1995) have shown that the directional tuning of hippocampal place cells codes for a planned trajectory between points of special significance, and that the place cells have episodic characteristics; the place fields being stable under constant task parameters, but the fields change when task parameters change. Wood et al. (2000) recorded cellular responses from dorsal CA1 pyramidal cells, which had place fields on the central stem of a T-maze, while rats performed a continuous spatial delayed alternation task. Approximately 70% of these cells fired differentially on left turn and right turn trials. Frank et al. (2000) also recorded from dorsal CA1 pyramidal cells, which had place fields on the central arm of a W-track, while rats performed a continuous spatial alternation task. Many of these neurons showed either prospective or retrospective coding, firing at different rates depending on where the rat would go (prospective) or where it had just come from (retrospective). They suggest that prefrontal inputs to entorhinal cortex and hippocampus mediate the prospective coding.

Ferbinteanu and Shapiro (2003), recording from CA1 pyramidal cells during a T-maze task in rats, were able to classify some cells as typical place cells. However, many of pyramidal cells had prospective coding, signaling response intention (e.g., the firing of these cells in the start arm of the maze predicted the subsequent goal arm choice). Yet other cells had retrospective coding, firing in the goal arm at the completion of the path traveled. Allen et al. (2012) tested rats on a continuous alternation task in a T-maze. They found that left and right choices were encoded by changes in the firing rates of hippocampal place cells, even though the actual place field remained constant. Importantly, they found that firing rate was an excellent predictor of response choice on correct trials, but not on error trials. They suggest a dual role of the hippocampus on this task, providing both allocentric spatial information and task contingent information supporting the rats' behavior. Ainge et al. (2012) tested rats on a conditional discrimination in a T-maze. Their results suggested that the firing of hippocampal CA1 place cells was not controlled by the conditional stimulus, but rather by the intended trajectory of the animal.

Song et al. (2005) recorded neural activity from dorsal hippocampal CA1/CA3 cells while rats either actively ran or passively rode in a motorized cart on a circular track. The spatial informational content of place cells was significantly lower under passive compared to active movement, even though the external sensory inputs were virtually identical in both conditions. They suggest that the better spatial resolution during active movement may be related to motor commands, efference copy, and proprioceptive feedback along with the concomitant involvement of the prefrontal cortex. Similar findings were reported by Terrazas et al. (2005). These findings are reminiscent of the results reported in humans by Voss et al. (2011), described earlier, showing that volitional control improves memory.

Dayawansa et al. (2006) recorded unit activity from CA1 place cells while rats moved on a motion stage along two routes in a Figure-8 pattern. In some cases the rats ran on a treadmill attached to the motion stage, in the same direction and at the same rate as the motion stage. In other cases, such locomotion on the treadmill was not allowed. They found that 85.2% of place cells, with place fields on the central stem, showed different firing patterns for the two routes when the rats were allowed to run on the treadmill. However, such route-dependent neural activity was lost when the treadmill was stopped (preventing running), even though the motion stage continued its movement along the routes. Thus, the route-correlated activity was locomotion dependent.

Clearly, these results support the critical role of movement for the activity of hippocampal pyramidal cells. Moreover, the firing of these cells appears to be episodic in nature, and correlates with response intention and perhaps response outcomes.

It may be that the hippocampus plays important roles in both pure spatial mapping and for the encoding of movement trajectories through the mapped space. Rondi-Reig et al. (2006) tested mice on a water star-maze task. Normal mice used either (or both) allocentric and sequential egocentric (multiple body turns) strategies to solve the task. Mice with knockout of the gene for the NR1 subunit of the NMDA receptor in the CA1 region of the hippocampus were impaired in using both strategies, and the impairment could not be explained by impairments of response inhibition. Interestingly, the knockout mice could acquire a simple egocentric strategy of one body turn. Perhaps anterior cortex-hippocampal relations mediate sequential egocentric strategies, posterior cortex-hippocampal relations mediate allocentric strategies, and cortico-striatal relations mediate simple egocentric strategies (see also Numan, 2000; Jacobs and Schenk, 2003; Griffiths et al., 2014).

## Theta, hippocampus, and prefrontal cortex

The hippocampal theta rhythm is a rhythmic sinusoidal EEG activity with a frequency range between 3 and 12 Hz (Bland, 2000). The GABAergic and cholinergic projections form the MSDB to the hippocampus play a critical role in the regulation of this rhythm (Lewis and Shute, 1967; Amaral and Kurz, 1985; Bland, 2000). Damage to the MSDB disrupts the hippocampal theta rhythm (Green and Arduini, 1954; Donovick, 1968; Bland, 1986, 2000) and results in behavioral impairments similar to those produced by direct damage to the hippocampus (Numan, 1978; Olton et al., 1982; Givens and Olton, 1990; Walsh, 2000; Pang et al., 2011). Hence, the integrity of the circuitry that maintains the theta rhythm appears to be critical for the normal function of the hippocampus. There have been many hypotheses about the behavioral correlates of the hippocampal theta rhythm, including attention, information processing, sensory-motor integration, voluntary movement, match/mismatch detection, and memory (see Vanderwolf, 1971; Bland, 2000; Hasselmo, 2000; Vinogradova, 2001; Buzsáki, 2002, 2005 for reviews). Buzsáki (2005) concludes that theta “is the temporal means of navigation in both neuronal space during episodic memory and real space during self-motion.”

Importantly, for our discussion, a number of reports have linked the theta rhythm to hippocampal-prefrontal interactions. Jones and Wilson (2005) recorded hippocampal theta activity as well as unit responses in dorsal CA1 and medial prefrontal cortex (mPFC) while rats performed a continuous alternation task. They found that unit responses in both CA1 and medial prefrontal cortex were phase locked to the theta rhythm, suggesting a neuronal interaction between the hippocampus and prefrontal cortex. Importantly, they also found that correlated unit spike timing, theta phase locking, and theta coherence between the dorsal CA1 and mPFC were strongest during the working memory component of the task, especially when the end result was a correct response. These data suggest that better communication between mPFC and dorsal hippocampus correlates with better working memory. They also suggest, since the mPFC receives input from ventral CA1/subiculum, that the dorsal CA1 interacts with the mPFC via the ventral CA1/subiculum. This idea is supported by the findings O'Neill et al. (2013) described earlier.

Siapas et al. (2005) reported findings similar to those reported by Jones and Wilson (2005). In further support, Benchenane et al. (2010) studied theta coherence between medial prefrontal cortex and hippocampus in rats during a Y-maze task. The theta coherence increased at the maze choice point and the greater the coherence, the better the performance. Carr et al. (2011), also studying rats, found that theta coherence between the hippocampus and prefrontal cortex is greatest at locations where the animals executed memory-guided decisions.

Hyman et al. (2011), testing rats on a DNMS (Delayed Non-Match to Sample) lever-press task, found that the firing of some neurons in rat medial prefrontal cortex become entrained to the hippocampal theta rhythm (theta cells), while others (about 1/3) did not (never theta cells). The theta cells appeared to be related to correct performance, as theta entrainment occurred for correct choices. In contrast the never theta cells appeared to predict errors as their response rates increased for incorrect choices.

Cardoso-Cruz et al. (2013), using partial directed coherence in rats tested on a working memory task, found bi-directional flow of information between the medial prefrontal cortex and dorsal CA1, at the theta frequency band, when the rat is in the choice zone of the maze. Additionally, most prefrontal neurons increased their firing rates in the choice zone when the rat performed a correct response. In contrast the CA1 neurons decreased their activity in the choice zone when the rat performed an error response.

Kaplan et al. (2012) tested human male participants as they pressed a key to move around a virtual environment and pick up objects along the way. Once well learned, they were shown an object cue, and now their task was to press the key to navigate to the remembered location of that object in the virtual environment. Using MEG and fMRI, they found a significant increase in theta power, in both the medial prefrontal cortex and the hippocampus, with movement onset compared to stationary periods. They also found a significant memory-related increase in theta power during object cue presentation; theta power at that time being greater for accurate compared inaccurate location choices. Also in humans, Anderson et al. (2010) used intracranial electroencephalogram to detect theta coherence between the lateral prefrontal cortex and the medial temporal lobe during the recall of a list of words. During recall, the theta coherence between the lateral prefrontal cortex and the medial temporal lobe was significantly greater than during a control baseline procedure. Granger causality analysis indicated a bidirectional flow of information between the lateral prefrontal cortex and the medial temporal lobe, but with a greater influence from the medial temporal lobe to the lateral prefrontal cortex.

These findings are relevant because the synchronization of oscillatory phases between different brain regions appears to facilitate both working memory and long term memory processes (Fell and Axmacher, 2011). Moreover, Sauseng et al. (2010) suggest that prefrontal theta coupled with gamma phase in other brain regions might provide a comparator function during working memory processes.

Taken together, these results support an important role for the theta rhythm in mediating interactions between the prefrontal cortex and hippocampus, and perhaps in the strengthening of correct actions and the weakening of incorrect actions. This conclusion is strengthened by data showing that tetanic stimulation of the hippocampus in rats induces LTP in the medial prefrontal cortex while low frequency stimulation of the hippocampus induces LTD or depotentiation (reversal of LTP) in the medial prefrontal cortex (Laroche et al., 2000; Vertes, 2006).

## Episodic memory

Wheeler et al. (1997) review a large body of data which argue that episodic memory depends upon autonoetic consciousness, which is the capacity to mentally represent and become aware of the self and its personal, subjective experiences across time: the present, past and future. Their review also supports the view that the prefrontal cortex is the primary brain region responsible for autonoetic consciousness, and hence essential for episodic memory.

While Wheeler et al. (1997) believe that autonoetic consciousness, and hence episodic memory, is a distinctly human capacity, our view is that the corollary discharge from prefrontal cortex to hippocampus, in non-human animal species, is the evolutionary precursor for autonoetic consciousness, allowing for episodic-like memory. Whishaw and Wallace (2003) agree, arguing that self-movement cues (including corollary discharge) in non-human animal species may be the antecedent to episodic memory. They suggest that “it is likely that self-movement information provides the animal with information about what it itself did at a particular time and place just as autobiographical memory allows humans to know what they did at a particular time and place.” Crystal (2010, 2012), Eacott and Easton (2012) and Martin-Ordas and Call (2013) review a large body of data supporting both episodic-like memory and prospective cognition in non-human animal species. Importantly, Veyrac et al. (2015) report that episodic-like memory in rats depends upon an intact hippocampus, and that the recollection of such memories correlates with hippocampal-prefrontal activation.

A number of studies have supported an interaction between the prefrontal cortex and the medial temporal lobe during episodic memory and prospective cognition in humans. Buckner and Carroll (2007) argue that episodic memory is involved in the projection of the self into the past (retrospective episodic memory) or the future (prospective cognition), and is mediated by an interaction between the prefrontal cortex and the medial temporal lobe (also see Simons and Spiers, 2003; Schacter et al., 2012). Botzung et al. (2008) used fMRI to study brain activation while human participants retrieved past episodic memories or while they planned future projects. Both the memory task and the future planning task activated the dorsolateral prefrontal cortex, the medial prefrontal cortex, and the hippocampus. As already noted, Anderson et al. (2010) found that theta coherence between the lateral prefrontal cortex and the medial temporal lobe increased during memory recall in humans.

Gerlach et al. (2011) used fMRI to study brain activation while human participants mentally simulated how they would solve a personal problem. This was a goal directed task that involved both self-referential and planning processes. They point out that the default network, which includes many structures including the medial temporal lobe, the medial prefrontal cortex, and the posterior cingulate cortex, is usually active during self-referential thought. In contrast, the dorsal attention network, which includes many structures including the dorsolateral prefrontal cortex, is active during focused attention. Usually, activity in these two networks is anticorrelated (also see Spreng et al., 2010). However, for their mentally simulated personal problem solving task, both networks were activated, including dorsolateral prefrontal cortex, medial prefrontal cortex, posterior cingulate cortex, and hippocampus. Moreover, functional connectivity analysis found strong connectivity between the dorsolateral prefrontal cortex and the posterior cingulate cortex. Therefore, the posterior cingulate, a component of retrosplenial cortex, may be a critical hub between the dorsolateral prefrontal cortex and the hippocampus (Vann et al., 2009; Fornito et al., 2012; Schacter et al., 2012; Mizumori and Jo, 2013; Miller et al., 2014).

Kim (2012) used a meta-analysis of imaging studies to support the view that autobiographical memory involves a self-referential component and an episodic memory component. He developed a dual system concept whereby the self-referential component involves the anteromedial prefrontal cortex and the memory component involves the medial temporal lobe.

These findings can be integrated with Numan's (1978) views by suggesting that a planned act of personal doing sends an efference copy from the dorsolateral prefrontal cortex to hippocampal area CA3 (the episodic memory component). When the action is initiated (along with increased theta coherence between prefrontal cortex and hippocampus), dorsal hippocampal area CA3 transfers the efference copy to dorsal hippocampal area CA1 (our comparator). When dorsal CA1 receives the efference copy, it activates ventral hippocampal area CA1 (Penley et al., 2013; Yang et al., 2014), which in turn activates the ventromedial prefrontal cortex which provides the self-referential component. This possibility makes sense if we assume that self-referential processes involve an affective component mediated by the ventral hippocampus and ventromedial prefrontal cortex. Alternatively, dorsolateral prefrontal cortex might activate the ventromedial prefrontal cortex at the same time that it sends the corollary discharge to hippocampus. This latter alternative receives some support from a recent fMRI study in humans by Hare et al. (2014). These authors, using Dynamic Causal Modeling, found increased effective connectivity from the left dorsolateral prefrontal cortex (dlPFC) to the ventromedial prefrontal cortex (vmPFC) during decisions to wait for larger delayed rewards in a temporal discounting task. They conclude that the influence of the dlPFC on the vmPFC is critical for effective self-control during goal-directed behavior.

## The medial septum-diagonal band

If the hippocampus serves as a response intention-response outcome working memory comparator, and if the comparator is enabled by the hippocampal theta rhythm, then damage to the MSDB which abolishes the theta rhythm, should also disable the comparator.

The MSDB projects cholinergic, GABAergic and glutamatergic terminals to the hippocampus (Roland et al., 2014) and is a critical subcortical structure for the pacing of the hippocampal theta rhythm (Bland, 2000; Buzsáki, 2002; Goutagny et al., 2008). Damage to the MSDB abolishes the hippocampal theta rhythm and results in behavioral impairments similar to those observed following direct damage to the hippocampus (see Numan, 1978, 2000; Gray, 1982 for reviews). Hence, the functions of the MSDB and hippocampus are intimately interrelated. Numan (1972, 1978, 2000) hypothesized that the pacing of the hippocampal theta rhythm by the MSDB enabled the hippocampal response intention-response outcome working memory comparator. Hence, damage to MSDB would disable the hippocampal comparator, and mimic many of the behavioral effects of direct damage to the hippocampus. A hallmark of septohippocampal damage is response perseveration. The failure of the response intention-response outcome working memory comparator would prevent the strengthening of correct responses (match) and the weakening of incorrect responses (mismatch). Both of these failures would lead to increased response perseveration errors compared to control subjects with an intact comparator system.

Numan and Quaranta (1990) assessed the effects of MSDB electrolytic lesions in rats on a delayed alternation (DA) task conducted in operant chambers with left and right levers. All testing was conducted post-operatively using three delay conditions (0, 10, and 20 s). MSDB lesions did not impair performance at the 0-s delay, indicating that the rats acquired the basic task rules, and that procedural/reference memory was intact. However, the MSDB lesioned rats were significantly impaired at the 10 and 20-s delays, indicating an impairment in working memory. The rats were then maintained at the 20-s delay, but a cue light was continuously illuminated above the left lever in order to reduce the spatial requirement of the task. This cue condition did not improve the performance of the MSDB lesioned rats, and they remained severely impaired compared to the sham-operated controls. However, in the next phase of the experiment, when a cue light was illuminated above the correct lever at the end of each 20-s delay (which eliminated the working memory requirement of the task), the MSDB lesioned rats dramatically improved their performance, which was now indistinguishable from the sham-operated controls.

The MSDB lesioned rats did not emit more perseverative errors compared to controls during the 0-s delay or during the 20-s delay when a cue light signaled the correct lever response. Hence, perseveration did not occur when working memory was not required. In contrast, the MSDB lesioned rats did emit significantly more perseverative errors than controls during the 10 and 20-s delay phases, as well as during the 20-s delay phase where a cue light was continuously illuminated above the left lever. Hence, perseveration only occurred in the MSDB lesioned rats when working memory was required. We then removed all perseverative errors from our data analysis (for both MSDB lesioned and control rats), counting only initial errors. The MSDB rats were still impaired, compared to controls, during the working memory phases of the task at both the 10 and 20-s delays. Taken together, these results suggest that a working memory impairment, rather than response perseveration, is the primary effect of the MSDB lesion. Hence, perseveration is a secondary consequence of the working memory failure.

Since the MSDB lesioned rats acquired the alternation rule, as indicated by their performance at the 0-s delay, then the impairment produced by adding a delay suggests a working memory failure for either their pre-delay response or their planned post-delay response. Hence the probability of repeating the same response following the delay would approximate 50%. This would result in an increase in both initial errors as well as perseverative errors when compared to control subjects. This analysis suggests, therefore, that perseverative errors are no different from initial errors: they are both due to a failure of working memory, leading to a random selection of lever responses following the delay which leads to an increased probability (compared to controls with intact working memory) of emitting the same response.

Finally, the two cue conditions, one which reduced the spatial requirement of the task but did not improve performance of the MSDB lesioned rats, and the other which reduced the working memory requirement of the task and did improve the performance of the lesioned rats, argue that the lesion disrupted some aspect of working memory, and that neither the spatial aspect of the task nor a failure of response inhibition were essential factors for the observed impairment.

In a subsequent experiment, Numan (1991) showed that MSDB lesioned rats are impaired on a delayed lever alternation task even after they acquired efficient delay performance preoperatively. This finding further supports a lesion induced failure of working memory, as task performance should be impaired irrespective of preoperative experience, so long as task performance is working memory dependent.

Since two levers (left and right) were employed in our delayed alternation experiments, it might be argued that a lesion induced spatial memory impairment could not be entirely ruled out. Moreover, since our hypothesis proposes a specific failure of the working memory for voluntary responses, we still needed to determine if other types of working memory remain intact after MSDB lesions. Therefore, we conducted two go/no-go tasks in operant chambers using only a single lever. The use of only one lever eliminated the left-right spatial component of the delayed alternation experiments described above. Both experiments included a non-delay condition (to assess procedural/reference memory) and a delay condition (to assess working memory). In the first experiment (Numan and Klis, 1992), the effects of MSDB lesions on stimulus working memory were assessed, whereas the effects of these lesions on response working memory were determined in the second experiment (Numan et al., 1995).

In the first experiment (Numan and Klis, 1992) a 2800 Hz tone and a 10 Hz flashing light served as “go” and “no-go” stimuli, respectively. A discrete trial procedure with symmetrical reinforcement was employed. At the beginning of each test session the chamber was dark except for back-illumination of a central press panel. Depression of the panel extinguished its back light and initiated the random presentation of either the “go” stimulus (tone) or the “no-go” stimulus (flashing light) for 3 s, after which the stimulus was terminated and the delay period was initiated. At the end of the delay, a white cue lamp was turned on above the lever for 2 s to indicate its functional availability for only 2 s. If, during this 2-s period, the rat pressed the lever on “go” trials or refrained from pressing it on “no-go” trials, a food pellet was delivered. At the end of the 2-s lever availability period, or following a lever press, the lever cue lamp was extinguished, and the central press panel was again back-illuminated to begin the next trial.

MSDB lesioned and sham-operated control rats were tested for 45 sessions, first using a 0-s delay (20 sessions) followed by 25 sessions employing an 8-s delay. The groups did not differ at the 0-s delay, both performing at about 90% correct during the last 5 days of testing. These results indicate intact reference/procedural memory in the MSDB lesioned rats. When the delay phase was initiated, performance fell to chance levels in both groups, but they improved over the 25 day testing period. However, in this external cue working memory task the MSDB lesioned rats performed significantly better that the sham-operated controls. During the last 5 days of the 8-s delay phase, the MSDB lesioned rats averaged 71% correct responding and the controls averaged 61% correct (*p* < 0.001).

These results cannot be explained by spatial theories of septohippocampal function (only one lever was used and discrete exteroceptive cues signaled the appropriate response) nor by a general process working memory impairment produced by the MSDB lesion (as the lesion facilitated performance during the working memory component of the task). Since our hypothesis proposes that the MSDB lesions result in impaired working memory for voluntary responses, it is possible that such an impairment would result in a compensatory reliance on exteroceptive cues to guide behavior, and hence facilitated performance, compared to controls, on this stimulus based working memory task. If this view is correct, then a go/no-go task that depends on response working memory, rather than stimulus working memory, should be impaired by MSDB lesions.

Numan et al. (1995) tested MSDB lesioned and sham-operated rats on a single lever operant go/no-go task similar to the one just described. However, external stimuli were not employed to signal the correct response, rather the rats were simply required to alternate “go” and “no-go” responses, first at a 0-s delay (20 sessions) and then at a 15-s delay (35 sessions). Again, a discrete trial procedure with symmetrical reinforcement was used.

In order to perform well on this task, the rat must acquire the go/no-go alternation rule and remember the response it emits on any given trial and alternate that response on the subsequent trial. Hence, the delay phase places a specific demand on response working memory (either holding the pre-delay response or the planned post-delay response in working memory).

Both the lesioned and control rats efficiently (>80% correct) acquired the task at the 0-s delay, and their performance did not significantly differ. These results suggest intact reference/procedural memory. In contrast, the MSDB rats were significantly impaired, compared to controls, during the 15-s delay phase of this task. Of particular interest is the dissociation of the effects of the MSDB lesions on the two versions of the delayed go/no-go task. When salient exteroceptive cues signaled the “go” and “no-go” trials, the MSDB lesioned rats performed better than controls. When such salient cues were lacking, however, and performance depended on response working memory, the MSDB lesioned rats were impaired. Clearly, these results do not support a general process working memory impairment following MSDB lesions. Rather, the impairment is specific for the working memory of voluntary responses, and can be expressed independently from spatial factors or response perseveration *per se*.

Next we assessed if damage to the MSDB would impair the rat's ability to distinguish between response-dependent and response-independent reinforcement under conditions of a working memory load. In that experiment (Numan et al., 2004), MSDB lesioned rats and sham-operated controls were tested on a single lever operant task to assess the effects of non-contingent reinforcement (response independent food pellet delivery) on response rates for contingent reinforcement (response dependent food pellet delivery). MSDB lesioned rats performed identically to controls under conditions of immediate contingent reinforcement (food pellet delivery immediately follows the lever press). Like control rats, they decreased their response rates for contingent reinforcements as the probability of non-contingent reinforcement increased. This normal action-outcome association is probably mediated by the striatum (Humphries and Prescott, 2010).

In contrast, when contingent reinforcement was delayed by 5-s (requiring working memory to bridge the response-reinforcement interval), the MSDB lesioned rats continued to press the lever for contingent reinforcement even as the probability of non-contingent reinforcement increased. Conversely, under these conditions of delayed contingent reinforcement, the control subjects remained sensitive to the increased probability of non-contingent reinforcement, and decreased their response rates for contingent reinforcement. It should be emphasized that this lesion effect was not due to the delay, *per se*. We found that under conditions of a high probability of delayed contingent reinforcement, but in the absence of non-contingent reinforcement presentations, the performance of the sham and septal lesioned rats was virtually identical. This ability to make action-outcome associations under conditions of delayed contingent reinforcement is also probably mediated by the striatum, especially the nucleus accumbens core (Cardinal, 2006).

Thus, the impaired sensitivity to the effects of non-contingent reinforcement presentations, evidenced by the MSDB lesioned rats, occurred only when *both* delayed contingent reinforcement and the presentation of non-contingent reinforcement occurred together; either alone did not differentially affect the MSDB lesioned rats.

Therefore, we concluded that these results reflected a failure of voluntary response working memory in the MSDB lesioned rats. This memory failure produced ambiguity between contingent (response-dependent) and non-contingent (response independent) reinforcement only when the contingent reinforcements were delayed, reducing the sensitivity of the MSDB lesioned rats to the response suppressing effects of the non-contingent reinforcements under these delay conditions.

Our views are not meant to argue against spatial (O'Keefe and Nadel, 1978) or relational (Eichenbaum and Cohen, 2001) theories of septohippocampal function, but rather to complement them, suggesting that one component of such learning-memory systems involves a voluntary response working memory system modulated, in part, by relations between the prefrontal cortex, hippocampus, and MSDB.

## Plausible mechanisms

Our hypothesis has focused on the interaction between the prefrontal cortex and hippocampus for the encoding of the memory for goal-directed voluntary actions, and that this “action memory” is an essential component of episodic memory. We argue that the prefrontal cortex develops the action plan and transmits an efference copy (corollary discharge) to the hippocampus. This corollary discharge provides the self-referential component to the memory, making it episodic.

We also argue that this “action memory” is strengthened (consolidated) or modified (behavioral/cognitive flexibility) by interactions between a hippocampal comparator and prefrontal cortex. We have presented evidence that the dorsal CA1 region of the hippocampus functions as a response intention-response outcome working memory comparator, and that the comparator is enabled by the hippocampal theta rhythm. When response outcomes *match* the response intention, the current action plan is strengthened; when there is a *mismatch* between the response intention and the actual response outcomes, the current action plan is weakened and a new action plan is formulated.

We hypothesize that an intention to act (based on an action plan) is mediated by the dorsolateral prefrontal cortex and its anatomical relations with cortical motor regions (Paus, 2001; Lau et al., 2004; Miyachi et al., 2005) and that the memory of that intention (the corollary discharge) is stored in the hippocampus (Belchior et al., 2014).

The dorsolateral prefrontal cortex transmits the efference copy (corollary discharge) to dorsal hippocampal area CA3. The activation of the response intention (preparation for an overt behavioral response) transfers the efference copy to dorsal hippocampal area CA1 (our comparator) and increases theta power in the hippocampus (Belchior et al., 2014). It is possible that this theta activation is mediated by projections from dorsal CA1 to the dorsomedial region of the lateral septum, which in turn projects to the theta control regions of the MSDB and supramammillary nucleus (Fanselow and Dong, 2010; see also Numan and Lubar, 1974). During new learning, this increased baseline theta power, across the dorso-ventral axis of the hippocampus (Penley et al., 2013; Yang et al., 2014; Long et al., 2015), promotes increased theta coherence between ventral CA1/subiculum and medial prefrontal cortex to strengthen the *new learning* (Benchenane et al., 2010), and perhaps to instantiate the self-referential tag for the new learning (Philippi et al., 2012).

We propose that this increased theta power during new learning serves as a baseline, and that changes from this baseline theta power can signal subsequent comparator outcomes in dorsal CA1. Here, the efference copy (response intention memory) arrives over the trisynaptic path (entorhinal cortex > dentate gyrus > CA3 > CA1), and when the action is initiated, the actual response outcomes are transmitted over the direct path from entorhinal cortex > CA1. These two inputs are compared in dorsal CA1. Recall that Zou et al. (2009) and Aitake et al. (2011) found that when there was a mismatch between response intention and response outcome, dorsal hippocampal theta power increased above baseline levels. Perhaps this increased theta power serves as a mismatch signal to facilitate behavioral switching. In support of this idea, Schmidt et al. (2013) found an increase in theta power in dorsal hippocampus when rats were required to shift a behavioral strategy in a + maze task. Moreover, Penley et al. (2013) found that both theta power and coherence increased in hippocampal area CA1, between electrode sites across the entire dorso-ventral axis of the hippocampus, when rats traversed a maze that was different from a maze they were previously exposed to. These findings suggest the functional integration of this associative mismatch signal across the dorso-ventral axis of the hippocampus. If so, this mismatch signal can be transmitted from ventral CA1/subiculum, over the known monosynaptic path, to the medial prefrontal cortex, and perhaps also instantiate a self-referential tag. But, how can the action plan now be modified in in dorsolateral prefrontal cortex? Cavanagh et al. (2009), testing human subjects on a Flanker task, found that theta power increased in medial prefrontal cortex immediately after an error, and that theta phase synchronization between medial prefrontal cortex and dorsolateral prefrontal cortex also dramatically increased on these error trials. The degree of these power and synchronization changes predicted the subsequent behavioral adjustments.

Clearly, our analysis is speculative, and much additional research is necessary to strengthen our position. Other brain regions also produce error signals, such as the ventral tegmental area (Schultz, 2002) and the anterior cingulate cortex (Holroyd and Coles, 2002). How our proposed mechanisms interact with these brain regions to foster behavioral flexibility and episodic memory remains to be determined. What is crucial to our hypothesis is that the fronto-hippocampal comparator is only essential for behavioral flexibility and episodic memory under conditions of a working memory load when salient external stimuli are not reliably available to guide behavior. When the working memory load is minimal, and /or when salient stimuli are available to guide responding, other neural systems can effectively regulate the learning and memory of these stimulus-response associations (van der Meer et al., 2012). If, as our hypothesis suggests, increases in hippocampal theta power reflect the engagement of our comparator during hippocampal dependent learning, then such power changes should not occur on hippocampal independent tasks. In support, Olvera-Cortés et al. (2002) and Sakimoto et al. (2013) found increases in hippocampal theta power when rats were tested on a hippocampal dependent task, but not when tested on a hippocampal independent task.

## Discussion

**Figure 1:**
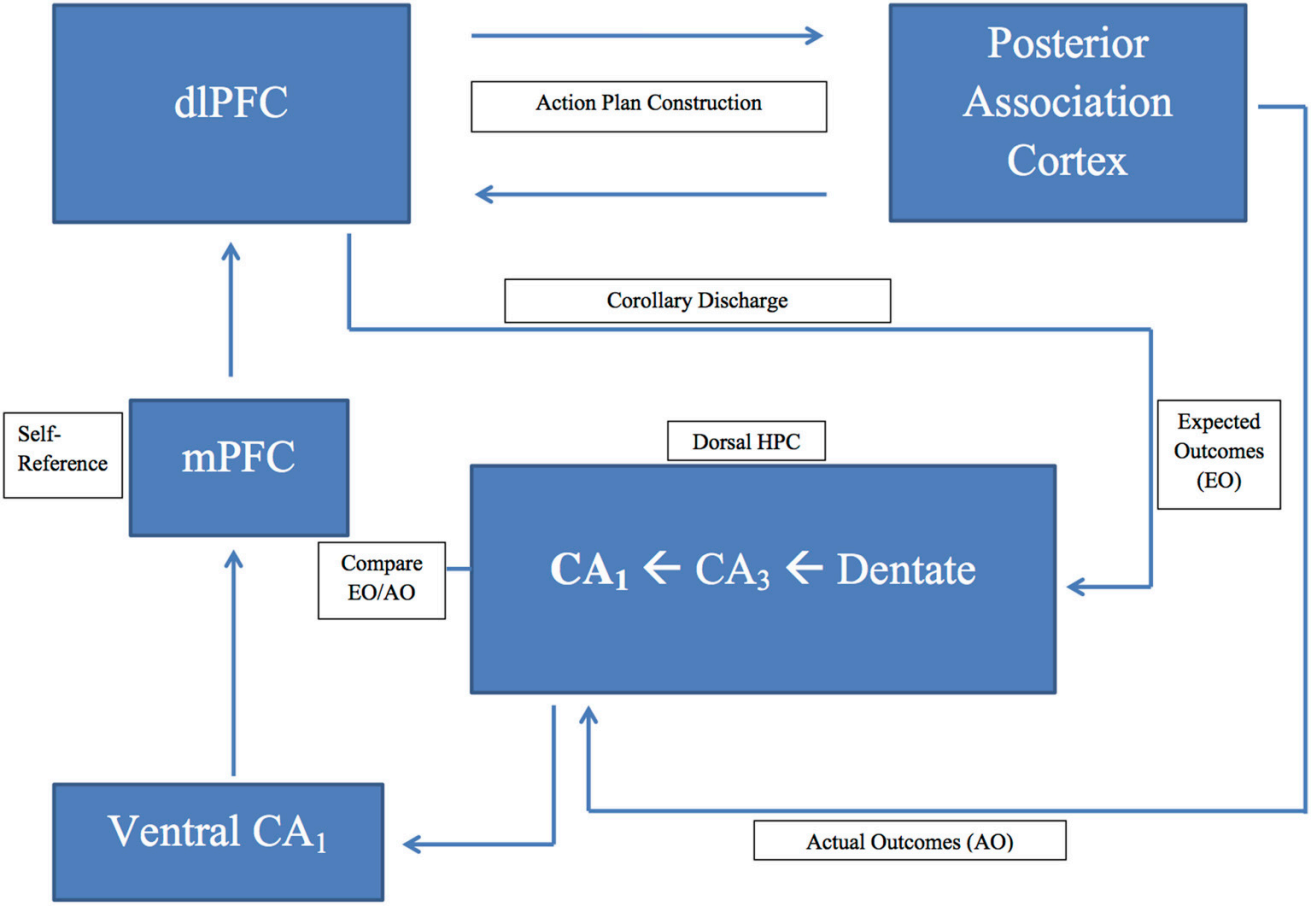
**A schematic diagram of the prefrontal-hippocampal comparator model presented in the text; dlPFC, dorsolateral prefrontal cortex; HPC, hippocampus; mPFC, medial prefrontal cortex**.

Our hypothesis integrates a large body of data supporting the view that a prefrontal-hippocampal comparator system facilitates the formation of episodic memory and its self-referential tag, and fosters behavioral flexibility and memory updating during goal-directed behavior. Behavioral planning to achieve a goal, voluntary action toward that goal, corollary discharge, and an associative match/mismatch comparator are central components of our hypothesis (see Figure 1). The brain regions that play a central role in our hypothesis include the prefrontal cortex (behavioral planning, action activation, and self-referential tag), the hippocampus (action-outcome working memory comparator), and the medial septum diagonal band (pacing the hippocampal theta rhythm). The hippocampal theta rhythm facilitates neural interaction between the hippocampus and prefrontal cortex, and signals comparator outcomes. This neural system appears to be functionally relevant under conditions of a working memory load when external stimuli are not available to guide behavior. In this section, I integrate the major findings in support of this hypothesis. Table 1 summarizes the research findings supporting our analysis.

**Table 1:** **Summary of major proposals and experimental results supporting the hypothesis**.

**Proposal**	**Support**
CA1 serves as an associative match-mismatch comparator	Fyhn et al., 2002; Lisman and Grace, 2005; Kumaran and Maguire, 2006; Duncan et al., 2009
Voluntary movement strengthens episodic memory	Numan et al., 1995, 2004; Dayawansa et al., 2006; Voss et al., 2011; Allen et al., 2012; Crystal et al., 2013
Autonoetic awareness is mediated by prefrontal-hippocampal interactions	Wheeler et al., 1997; Buckner and Carroll, 2007; Botzung et al., 2008; Gerlach et al., 2011; Schacter et al., 2012; Veyrac et al., 2015
Ventral hippocampus may instantiate the self-referential tag in mPFC	Kelley et al., 2002; Goto and Grace, 2008; Philippi et al., 2012; O'Neill et al., 2013; Brown et al., 2014
Theta rhythm signals comparator activation and hippocampal-PFC interactions	Jones and Wilson, 2005; Siapas et al., 2005; Cavanagh et al., 2009; Zou et al., 2009; Benchenane et al., 2010; Aitake et al., 2011; Schmidt et al., 2013
Schizophrenia symptoms may be due to a failure of corollary discharge and PFC-hippocampal abnormalities	Feinberg, 1978; Frith and Done, 1988; Friston and Frith, 1995; Feinberg and Guazzelli, 1999; Fletcher et al., 1999; Ford and Mathalon, 2004; Wolf et al., 2007; Stephan et al., 2009; Whitford et al., 2012; Ledoux et al., 2013; Thakkar et al., 2015

### Hippocampal area CA1 serves as an associative match-mismatch comparator

The research reported by Fyhn et al. (2002), Lisman and Grace (2005) and Lever et al. (2010) in rats, and Kumaran and Maguire (2006) and Duncan et al. (2009) in humans support the view that hippocampal area CA1 functions as an associative match-mismatch comparator. In other words, the CA1 comparator does not respond to novelty *per se*, but rather to a novel event that deviates from previously acquired experience. Thus, the comparator compares expected behavioral outcomes, derived from prior memories for the present context, with the actual current behavioral outcomes. One would expect, however, that the comparator would also be functional during the initial learning trials of a completely novel experience, perhaps indicated by an increase in theta power, which would strengthen this new learning. As learning stabilizes, and the experiential outcomes remain the same (signaled by comparator matches), the maintenance of this baseline theta power would foster the consolidation of the episodic memory for this experience via theta coherence between the hippocampus and prefrontal cortex (Jones and Wilson, 2005; Siapas et al., 2005; Benchenane et al., 2010), and subsequently via reciprocal anatomical connections between the prefrontal cortex and other regions of association cortex (Numan, 1978; Frankland and Bontempi, 2005). Now, if the contextual experiential outcomes change (e.g., reversal learning, placing the escape platform in a new water maze location, etc.), expected outcomes will no longer match actual outcomes of behavior, and hippocampal theta power will increase over the baseline level, signaling a mismatch (Zou et al., 2009; Aitake et al., 2011; Schmidt et al., 2013). Again, via theta coherence between hippocampus and prefrontal cortex, and prefrontal relations to other regions of association cortex, behavioral flexibility (e.g., response switching) and memory updating will be fostered (Kroes and Fernández, 2012).

### Voluntary movement strengthens episodic memories for goal-directed behavior

The prefrontal cortex plays an essential role in the planning of behavior to obtain a goal (Numan, 1978; Miller and Cohen, 2001). Our hypothesis proposes that the prefrontal cortex interacts with cortical motor areas to organize and initiate goal-directed actions, and transmits a corollary discharge to the hippocampus. This efference copy is stored as a working memory in the hippocampus to serve as an action-outcome comparator. I propose that the actual “doing” of behavior, and the assessment of the outcomes that follow, play a significant role in goal-directed behavior, behavioral flexibility, and the strengthening of the episodic memory for the experience. The importance of such behavioral “doing” is supported by the research of Voss et al. (2011) who found that volitional control in humans improves memory, and that this effect depends upon an intact hippocampus and correlates with hippocampal-prefrontal interactions. This view finds further support from the work of Crystal et al. (2013), showing that hippocampal lesions in rats impair source memory derived from active voluntary movement toward a goal vs. passive placement at the goal. Moreover, in a non-spatial operant task, Numan et al. (2004) found that damage to the septohippocampal system in rats impaired their ability to distinguish contingent (response dependent) from non-contingent (response independent) reinforcement. We have also described a number of experiments supporting important movement-related correlates for hippocampal “place cells.” Foster et al. (1989) found excellent place specificity for “place cells” under conditions of active movement, but not during restraint. Song et al. (2005) and Dayawansa et al. (2006) found better spatial informational content, and route-dependent neural firing, respectively, in hippocampal “place cells” recorded in rats during active movement compared to passive movement, even though the external environmental cues were constant for the active and passive conditions. A number of other experiments reported that “place cell” firing predicts movement trajectories (Muller and Kubie, 1989; Wood et al., 2000; Allen et al., 2012).

### Episodic memory and autonoetic awareness

Many theories of episodic memory argue that episodic memory depends on autonoetic consciousness: the capacity to mentally represent the self across time and that autonoetic awareness depends upon the prefrontal cortex and its interaction with the hippocampus (Wheeler et al., 1997; Tulving, 2002; Buckner and Carroll, 2007; Schacter et al., 2012). We propose that the substrate for such autonoetic consciousness, mediating episodic memory in humans and episodic-like memory in animals, is the corollary discharge from prefrontal cortex to hippocampus. A number of studies, using fMRI in humans, found that the retrieval of episodic memories and even the mental simulation (thoughts about an action plan and its consequences) about solving a personal problem correlates with activation of the prefrontal cortex (both dlPFC and mPFC) and the hippocampus (Botzung et al., 2008; Gerlach et al., 2011). Importantly, Veyrac et al. (2015) reported that episodic-like memory in rats depends upon the hippocampus, and that the recollection of such memories correlates with hippocampal-prefrontal interactions. In humans, the medial prefrontal cortex appears to play a critical role in self-referential memory (Kelley et al., 2002) and lesions of the medial prefrontal cortex abolish this self-referential effect on memory (Philippi et al., 2012).

### The ventral hippocampus and the self-referential component of episodic memory

There is a dissociation of function along the dorsoventral (posterior-anterior in primates) axis of the hippocampus with the dorsal region mediating cognitive, spatial and working memory functions and the ventral region mediating various aspects of affective behavior (Moser and Moser, 1998; Bannerman et al., 1999, 2003; Fanselow and Dong, 2010). We propose that the contextual components of episodic memory are mediated by projections from dlPFC to dorsal hippocampus, and that the self-referential component of episodic memory (which likely has an affective component) is initiated by the corollary discharge and instantiated by interactions between dorsal hippocampus and ventral hippocampus, and the direct projection from ventral hippocampus to medial prefrontal cortex. This conceptualization is supported by the by the work of Goto and Grace (2008) in rats, O'Neill et al. (2013) in mice, and Brown et al. (2014) in humans, as well as the research described in Section Episodic Memory and Autonoetic Awareness.

### The hippocampal theta rhythm

The hippocampal theta rhythm is a local field potential of the hippocampus that correlates with memory functions, sensory-motor integration, and voluntary movement. We have given theta a critical role in our hypothesis. While theta may be recorded from the hippocampus under a variety of conditions, especially during voluntary movement, we propose that theta power increases when subjects are exposed to a hippocampal-dependent learning experience (Olvera-Cortés et al., 2002; Sakimoto et al., 2013) and that this increased theta power (which we refer to as baseline theta power) strengthens correct responses (comparator match) through hippocampal-prefrontal interactions. In contrast, error responses (comparator mismatch) cause a further increase (above baseline) in theta power, providing a mismatch signal to the prefrontal cortex fostering behavioral flexibility and memory updating. The MSDB is an important pacemaker for the hippocampal theta rhythm; damage to the MSDB disrupts theta and results in behavioral impairments similar to those produced by direct hippocampal damage. Jones and Wilson (2005) recorded both unit responses and theta waves from dorsal CA1 and mPFC while rats performed an alternation task. The unit responses from both regions were phase locked to theta, and this phase locking as well as theta coherence between CA1 and mPFC were strongest during the working memory component of the task. They suggest, as we have above (Section The Ventral Hippocampus and the Self-Referential Component of Episodic Memory), since the mPFC receives input from the ventral hippocampus, that the dorsal hippocampus interacts with the mPFC via the ventral hippocampus. In my laboratory, we found that MSDB lesions in rats, which presumably disrupted the hippocampal theta rhythm, impaired the working memory for voluntary actions (Numan et al., 1995) and diminished the ability of rats to discriminate response-dependent contingent reinforcement from response-independent non-contingent reinforcement (Numan et al., 2004).

### Schizophrenia

A fertile area for future investigation, in support of our hypothesis, comes from the study of schizophrenia patients. Research has indicated that: (a) some of the symptoms of schizophrenia can be interpreted as a failure of corollary discharge, (b) schizophrenia patients have episodic memory impairments, and (c) abnormalities in prefrontal cortex, hippocampus, and/or disconnection between these brain regions may mediate these findings. It has been hypothesized that a failure of corollary discharge, and its concomitant self-referential tag, could contribute to the positive symptoms of schizophrenia (e.g., delusions, hallucinations); the patient being unable to distinguish their self-generated thoughts and actions from those externally generated (Feinberg, 1978; Frith and Done, 1988; Feinberg and Guazzelli, 1999; Stephan et al., 2009; Whitford et al., 2012; Thakkar et al., 2015). Importantly, the failure of this self-referential tag appears related to a disruption of communication between prefrontal cortex and the temporal cortex/hippocampus (Frith and Done, 1988; Friston and Frith, 1995; Fletcher et al., 1999; Ford and Mathalon, 2004). Wolf et al. (2007) found that impaired episodic memory in schizophrenia patients correlated with disrupted connectivity between the dlPFC and temporal/parahippocampal regions. A number of reviews (Heckers, 2001; Boyer et al., 2007; Ranganath et al., 2008) also emphasize episodic memory impairments in schizophrenia and concomitant structural and functional impairments in hippocampus and in prefrontal-hippocampal relations. Importantly, Ledoux et al. (2013) reported impaired episodic memory and impaired navigation (in a virtual environment) in schizophrenia patients. These impairments were associated with abnormal activations (fMRI) in the left middle frontal gyrus and both the left and right posterior hippocampus.

### Conflict of interest statement

The author declares that the research was conducted in the absence of any commercial or financial relationships that could be construed as a potential conflict of interest.

## References

[B1] Aggleton J. P. (2012). Multiple anatomical systems embedded within the primate medial temporal lobe: implications for hippocampal function. Neurosci. Biobehav. Rev. 36, 1579–1596. 10.1016/j.neubiorev.2011.09.005 21964564

[B2] Aggleton J. P. Hunt P. R. Rawlins J. N. P. (1986). The effects of hippocampal lesions upon spatial and non-spatial tests of working memory. Behav. Brain Res. 19, 133–146. 10.1016/0166-4328(86)90011-2 3964405

[B3] Ainge J. A. Tamosiunaite M. Wörgötter F. Dudchenko P. A. (2012). Hippocampal place cells encode intended destination, and not a discriminative stimulus, in a conditional T−maze task. Hippocampus 22, 534–543. 10.1002/hipo.20919 21365712

[B4] Aitake M. Hori E. Matsumoto J. Umeno K. Fukuda M. Ono T.. (2011). Sensory mismatch induces autonomic responses associated with hippocampal theta waves in rats. Behav. Brain Res. 220, 244–253. 10.1016/j.bbr.2011.02.011 21316395

[B5] Allen K. Rawlins J. N. P. Bannerman D. M. Csicsvari J. (2012). Hippocampal place cells can encode multiple trial-dependent features through rate remapping. J. Neurosci. 32, 14752–14766. 10.1523/JNEUROSCI.6175-11.2012 23077060 PMC3531717

[B6] Amaral D. G. Kurz J. (1985). An analysis of the origins of the cholinergic and noncholinergic septal projections to the hippocampal formation of the rat. J. Comp. Neurol. 240, 37–59. 10.1002/cne.902400104 4056104

[B7] Anderson K. L. Rajagovindan R. Ghacibeh G. A. Meador K. J. Ding M. (2010). Theta oscillations mediate interaction between prefrontal cortex and medial temporal lobe in human memory. Cereb. Cortex, 20, 1604–1612. 10.1093/cercor/bhp223 19861635

[B8] Anokhin P. K. (1969). Cybernetics and the integrative activity of the brain, in A Handbook of Contemporary Soviet Psychology, eds Cole M. Maltzman I. (New York, NY: Basic Books), 830–856.

[B9] Bannerman D. M. Grubb M. Deacon R. M. J. Yee B. K. Feldon J. Rawlins J. N. P. (2003). Ventral hippocampal lesions affect anxiety but not spatial learning. Behav. Brain Res. 139, 197–213. 10.1016/S0166-4328(02)00268-1 12642189

[B10] Bannerman D. M. Yee B. K. Good M. A. Heupel M. J. Iversen S. D. Rawlins J. N. P. (1999). Double dissociation of function within the hippocampus: a comparison of dorsal, ventral, and complete hippocampal cytotoxic lesions. Behav. Neurosci. 113, 1170–1188. 10.1037/0735-7044.113.6.1170 10636297

[B11] Belchior H. Lopes-Dos-Santos V. Tort A. B. Ribeiro S. (2014). Increase in hippocampal theta oscillations during spatial decision making. Hippocampus 24, 693–702. 10.1002/hipo.22260 24520011 PMC4229028

[B12] Benchenane K. Peyrache A. Khamassi M. Tierney P. L. Gioanni Y. Battaglia F. P.. (2010). Coherent theta oscillations and reorganization of spike timing in the hippocampal-prefrontal network upon learning. Neuron 66, 921–936. 10.1016/j.neuron.2010.05.013 20620877

[B13] Benetti S. Mechelli A. Picchioni M. Broome M. Williams S. McGuire P. (2009). Functional integration between the posterior hippocampus and prefrontal cortex is impaired in both first episode schizophrenia and the at risk mental state. Brain 132, 2426–2436. 10.1093/brain/awp098 19420091

[B14] Bland B. H. (1986). The physiology and pharmacology of hippocampal formation theta rhythms. Progress Neurobiol. 26, 1–54. 10.1016/0301-0082(86)90019-5 2870537

[B15] Bland B. H. (2000). The medial septum: node of the ascending brainstem hippocampal synchronizing pathways, in The Behavioral Neuroscience of the Septal Region, ed Numan R. (New York, NY: Springer-Verlag), 115–145.

[B16] Botzung A. Denkova E. Manning L. (2008). Experiencing past and future personal events: functional neuroimaging evidence on the neural bases of mental time travel. Brain Cogn. 66, 202–212. 10.1016/j.bandc.2007.07.011 17881109

[B17] Boyer P. Phillips J. L. Rousseau F. L. Ilivitsky S. (2007). Hippocampal abnormalities and memory deficits: new evidence of a strong pathophysiological link in schizophrenia. Brain Res. Rev. 54, 92–112. 10.1016/j.brainresrev.2006.12.008 17306884

[B18] Brown T. I. Whiteman A. S. Aselcioglu I. Stern C. E. (2014). Structural differences in hippocampal and prefrontal gray matter volume support flexible context-dependent navigation ability. J. Neurosci. 34, 2314–2320. 10.1523/JNEUROSCI.2202-13.2014 24501370 PMC3913873

[B19] Buckner R. L. Carroll D. C. (2007). Self-projection and the brain. Trends Cogn. Sci. 11, 49–57. 10.1016/j.tics.2006.11.004 17188554

[B20] Bussey T. J. Clea Warburton E. Aggleton J. P. Muir J. L. (1998). Fornix lesions can facilitate acquisition of the transverse patterning task: a challenge for “configural” theories of hippocampal function. J. Neurosci. 18, 1622–1631. 9454867 10.1523/JNEUROSCI.18-04-01622.1998 PMC6792739

[B21] Buzsáki G. (2002). Theta oscillations in the hippocampus. Neuron 33, 325–340. 10.1016/S0896-6273(02)00586-X 11832222

[B22] Buzsáki G. (2005). Theta rhythm of navigation: link between path integration and landmark navigation, episodic and semantic memory. Hippocampus 15, 827–840. 10.1002/hipo.20113 16149082

[B23] Cardinal R. N. (2006). Neural systems implicated in delayed and probabilistic reinforcement. Neural Netw. 19, 1277–1301. 10.1016/j.neunet.2006.03.004 16938431

[B24] Cardoso-Cruz H. Lima D. Galhardo V. (2013). Impaired spatial memory performance in a rat model of neuropathic pain is associated with reduced hippocampus–prefrontal cortex connectivity. J. Neurosci. 33, 2465–2480. 10.1523/JNEUROSCI.5197-12.2013 23392675 PMC6619155

[B25] Carr M. F. Jadhav S. P. Frank L. M. (2011). Hippocampal replay in the awake state: a potential substrate for memory consolidation and retrieval. Nat. Neurosci. 14, 147–153. 10.1038/nn.2732 21270783 PMC3215304

[B26] Cavanagh J. F. Cohen M. X. Allen J. J. (2009). Prelude to and resolution of an error: EEG phase synchrony reveals cognitive control dynamics during action monitoring. J. Neurosci. 29, 98–105. 10.1523/JNEUROSCI.4137-08.2009 19129388 PMC2742325

[B27] Chen J. Olsen R. K. Preston A. R. Glover G. H. Wagner A. D. (2011). Associative retrieval processes in the human medial temporal lobe: hippocampal retrieval success and CA1 mismatch detection. Learn. Mem. 18, 523–528. 10.1101/lm.2135211 21775513 PMC3256570

[B28] Cho Y. H. Kesner R. P. (1995). Relational object association learning in rats with hippocampal lesions. Behav. Brain Res. 67, 91–98. 10.1016/0166-4328(94)00109-S 7748506

[B29] Crystal J. D. (2010). Episodic-like memory in animals. Behav. Brain Res. 215, 235–243. 10.1016/j.bbr.2010.03.005 20211205 PMC2907447

[B30] Crystal J. D. (2012). Prospective cognition in rats. Learn. Motiv. 43, 181–191. 10.1016/j.lmot.2012.05.006 23180886 PMC3501753

[B31] Crystal J. D. Alford W. T. Zhou W. Hohmann A. G. (2013). Source memory in the rat. Curr. Biol. 23, 387–391. 10.1016/j.cub.2013.01.023 23394830 PMC3595394

[B32] Dayawansa S. Kobayashi T. Hori E. Umeno K. Tazumi T. Ono T.. (2006). Conjunctive effects of reward and behavioral episodes on hippocampal place−differential neurons of rats on a mobile treadmill. Hippocampus 16, 586–595. 10.1002/hipo.20186 16685707

[B33] Donovick P. J. (1968). Effects of localized septal lesions on hippocampal EEG activity and behavior in rats. J. Comp. Physiol. Psychol. 66, 569–578. 10.1037/h0026514 5721486

[B34] Duncan K. Curtis C. Davachi L. (2009). Distinct memory signatures in the hippocampus: intentional states distinguish match and mismatch enhancement signals. J. Neurosci. 29, 131–139. 10.1523/JNEUROSCI.2998-08.2009 19129391 PMC2789241

[B35] Duncan K. Ketz N. Inati S. J. Davachi L. (2012). Evidence for area CA1 as a match/mismatch detector: a high−resolution fMRI study of the human hippocampus. Hippocampus 22, 389–398. 10.1002/hipo.20933 21484934 PMC3529001

[B36] Eacott M. J. Easton A. (2012). Remembering the past and thinking about the future: is it really about time? Learn. Motiv. 43, 200–208. 10.1016/j.lmot.2012.05.012

[B37] Eichenbaum H. Cohen N. J. (2001). From Conditioning to Conscious Recollection: Memory Systems of the Brain. New York, NY: Oxford University Press.

[B38] Eichenbaum H. Fagan A. Mathews P. Cohen N. J. (1988). Hippocampal system dysfunction and odor discrimination learning in rats: impairment or facilitation depending on representational demands. Behav. Neurosci. 102, 331–339. 10.1037/0735-7044.102.3.331 3395444

[B39] Ellen P. Butter J. (1969). External cue control of DRL performance in rats with septal lesions. Physiol. Behav. 4, 1–6. 10.1016/0031-9384(69)90003-1

[B40] Ennaceur A. Meliani K. (1992). A new one-trial test for neurobiological studies of memory in rats. III. Spatial vs. non-spatial working memory. Behav. Brain Res. 51, 83–92. 10.1016/S0166-4328(05)80315-8 1482548

[B41] Fanselow M. S. Dong H. W. (2010). Are the dorsal and ventral hippocampus functionally distinct structures? Neuron 65, 7–19. 10.1016/j.neuron.2009.11.031 20152109 PMC2822727

[B42] Feinberg I. (1978). Efference copy and corollary discharge: implications for thinking and its disorders. Schizophr. Bull. 4, 636–640. 10.1093/schbul/4.4.636 734369

[B43] Feinberg I. Guazzelli M. (1999). Schizophrenia–a disorder of the corollary discharge systems that integrate the motor systems of thought with the sensory systems of consciousness. Br. J. Psychiatr. 174, 196–204. 10.1192/bjp.174.3.196 10448443

[B44] Fell J. Axmacher N. (2011). The role of phase synchronization in memory processes. Nat. Rev. Neurosci. 12, 105–118. 10.1038/nrn2979 21248789

[B45] Ferbinteanu J. Shapiro M. L. (2003). Prospective and retrospective memory coding in the hippocampus. Neuron 40, 1227–1239. 10.1016/S0896-6273(03)00752-9 14687555

[B46] Ferbinteanu J. Shirvalkar P. Shapiro M. L. (2011). Memory modulates journey-dependent coding in the rat hippocampus. J. Neurosci. 31, 9135–9146. 10.1523/JNEUROSCI.1241-11.2011 21697365 PMC3136141

[B47] Fletcher P. McKenna P. J. Friston K. J. Frith C. D. Dolan R. J. (1999). Abnormal cingulate modulation of fronto-temporal connectivity in schizophrenia. Neuroimage 9, 337–342. 10.1006/nimg.1998.0411 10075903

[B48] Foerde K. Race E. Verfaellie M. Shohamy D. (2013). A role for the medial temporal lobe in feedback-driven learning: evidence from amnesia. J. Neurosci. 33, 5698–5704. 10.1523/JNEUROSCI.5217-12.2013 23536083 PMC3865542

[B49] Ford J. M. Mathalon D. H. (2004). Electrophysiological evidence of corollary discharge dysfunction in schizophrenia during talking and thinking. J. Psychiatr. Res. 38, 37–46. 10.1016/S0022-3956(03)00095-5 14690769

[B50] Fornito A. Harrison B. J. Zalesky A. Simons J. S. (2012). Competitive and cooperative dynamics of large-scale brain functional networks supporting recollection. Proc. Natl. Acad. Sci. U.S.A. 109, 12788–12793. 10.1073/pnas.1204185109 22807481 PMC3412011

[B51] Foster T. C. Castro C. A. McNaughton B. L. (1989). Spatial selectivity of rat hippocampal neurons: dependence on preparedness for movement. Science 244, 1580–1582. 10.1126/science.2740902 2740902

[B52] Frank L. M. Brown E. N. Wilson M. (2000). Trajectory encoding in the hippocampus and entorhinal cortex. Neuron 27, 169–178. 10.1016/S0896-6273(00)00018-0 10939340

[B53] Frankland P. W. Bontempi B. (2005). The organization of recent and remote memories. Nat. Rev. Neurosci. 6, 119–130. 10.1038/nrn1607 15685217

[B54] Friston K. J. Frith C. D. (1995). Schizophrenia: a disconnection syndrome. Clin. Neurosci. 3, 89–97. 7583624

[B55] Frith C. D. Done D. J. (1988). Towards a neuropsychology of schizophrenia. Br. J. Psychiatr. 153, 437–443. 10.1192/bjp.153.4.437 3074851

[B56] Fyhn M. Molden S. Hollup S. Moser M. B. Moser E. I. (2002). Hippocampal neurons responding to first-time dislocation of a target object. Neuron 35, 555–566. 10.1016/S0896-6273(02)00784-5 12165476

[B57] Gaesser B. Spreng R. N. McLelland V. C. Addis D. R. Schacter D. L. (2013). Imagining the future: evidence for a hippocampal contribution to constructive processing. Hippocampus 23, 1150–1161. 10.1002/hipo.22152 23749314 PMC3838494

[B58] Gaffan E. A. Bannerman D. M. Healey A. N. (2003). Learning associations between places and visual cues without learning to navigate: neither fornix nor entorhinal cortex is required. Hippocampus 13, 445–460. 10.1002/hipo.10066 12836914

[B59] Gaffan E. A. Bannerman D. M. Warburton E. C. Aggleton J. P. (2001). Rats' processing of visual scenes: effects of lesions to fornix, anterior thalamus, mamillary nuclei or the retrohippocampal region. Behav. Brain Res. 121, 103–117. 10.1016/S0166-4328(00)00389-2 11275288

[B60] Gerlach K. D. Spreng R. N. Gilmore A. W. Schacter D. L. (2011). Solving future problems: default network and executive activity associated with goal-directed mental simulations. Neuroimage 55, 1816–1824. 10.1016/j.neuroimage.2011.01.030 21256228 PMC3855008

[B61] Givens B. S. Olton D. S. (1990). Cholinergic and GABAergic modulation of medial septal area: effect on working memory. Behav. Neurosci. 104, 849–855. 10.1037/0735-7044.104.6.849 2178347

[B62] Gothard K. M. Skaggs W. E. McNaughton B. L. (1996). Dynamics of mismatch correction in the hippocampal ensemble code for space: interaction between path integration and environmental cues. J. Neurosci. 16, 8027–8040. 8987829 10.1523/JNEUROSCI.16-24-08027.1996 PMC6579211

[B63] Goto Y. Grace A. A. (2008). Dopamine modulation of hippocampal–prefrontal cortical interaction drives memory-guided behavior. Cereb. Cortex 18, 1407–1414. 10.1093/cercor/bhm172 17934187 PMC2892381

[B64] Goutagny R. Manseau F. Jackson J. Danik M. Williams S. (2008). *In vitro* activation of the medial septum—Diagonal band complex generates atropine−sensitive and atropine−resistant hippocampal theta rhythm: an investigation using a complete septohippocampal preparation. Hippocampus 18, 531–535. 10.1002/hipo.20418 18306282

[B65] Granger R. Wiebe S. P. Taketani M. Lynch G. (1996). Distinct memory circuits composing the hippocampal region. Hippocampus 6, 567–578. 9034846 10.1002/(SICI)1098-1063(1996)6:6<567::AID-HIPO2>3.0.CO;2-E

[B66] Gray J. A. (1982). The Neuropsychology of Anxiety: An Enquiry into the Functions of the Septo-hippocampal System. New York, NY: Oxford University Press.

[B67] Green J. D. Arduini A. A. (1954). Hippocampal electrical activity in arousal. J. Neurophysiol. 17, 533–557. 13212425 10.1152/jn.1954.17.6.533

[B68] Griffin A. L. (2015). Role of the thalamic nucleus reuniens in mediating interactions between the hippocampus and medial prefrontal cortex during spatial working memory. Front. Syst. Neurosci. 9:29. 10.3389/fnsys.2015.00029 25805977 PMC4354269

[B69] Griffiths K. R. Morris R. W. Balleine B. W. (2014). Translational studies of goal-directed action as a framework for classifying deficits across psychiatric disorders. Front. Syst. Neurosci. 8:101. 10.3389/fnsys.2014.00101 24904322 PMC4033402

[B70] Hare T. A. Hakimi S. Rangel A. (2014). Activity in dlPFC and its effective connectivity to vmPFC are associated with temporal discounting. Front. Neurosci. 8:50. 10.3389/fnins.2014.00050 24672421 PMC3957025

[B71] Hasselmo M. E. (2000). Septal modulation of hippocampal dynamics: what is the function of the theta rhythm? in The Behavioral Neuroscience of the Septal Region, ed Numan R. (New York, NY: Springer-Verlag), 92–114.

[B72] Heckers S. (2001). Neuroimaging studies of the hippocampus in schizophrenia. Hippocampus 11, 520–528. 10.1002/hipo.1068 11732705

[B73] Hetherington P. A. Shapiro M. L. (1993). A simple network model simulates hippocampal place fields: II. Computing goal-directed trajectories and memory fields. Behav. Neurosci. 107, 434–443. 10.1037/0735-7044.107.3.434 8329133

[B74] Holroyd C. B. Coles M. G. (2002). The neural basis of human error processing: reinforcement learning, dopamine, and the error-related negativity. Psychol. Rev. 109, 679–709. 10.1037/0033-295X.109.4.679 12374324

[B75] Hudon C. Doré F. Y. Goulet S. (2003). Impaired performance of fornix-transected rats on a distal, but not on a proximal, version of the radial-arm maze cue task. Behav. Neurosci. 117, 1353–1362. 10.1037/0735-7044.117.6.1353 14674853

[B76] Humphries M. D. Prescott T. J. (2010). The ventral basal ganglia, a selection mechanism at the crossroads of space, strategy, and reward. Progr. Neurobiol. 90, 385–417. 10.1016/j.pneurobio.2009.11.003 19941931

[B77] Hyman J. M. Hasselmo M. E. Seamans J. K. (2011). What is the Functional Relevance of Prefrontal Cortex Entrainment to Hippocampal Theta Rhythms? Front. Neurosci. 5:24. 10.3389/fnins.2011.00024 21427795 PMC3052540

[B78] Jacobs L. F. Schenk F. (2003). Unpacking the cognitive map: the parallel map theory of hippocampal function. Psychol. Rev. 110, 285–315. 10.1037/0033-295X.110.2.285 12747525

[B79] Janisewicz A. M. Baxter M. G. (2003). Transfer effects and conditional learning in rats with selective lesions of medial septal/diagonal band cholinergic neurons. Behav. Neurosci. 117, 1342–1352. 10.1037/0735-7044.117.6.1342 14674852

[B80] Jay T. M. Witter M. P. (1991). Distribution of hippocampal CA1 and subicular efferents in the prefrontal cortex of the rat studied by means of anterograde transport of Phaseolus vulgaris−leucoagglutinin. J. Comp. Neurol. 313, 574–586. 10.1002/cne.903130404 1783682

[B81] Jones M. W. Wilson M. A. (2005). Theta rhythms coordinate hippocampal-prefrontal interactions in a spatial memory task. PLoS Biol. 3:e402. 10.1371/journal.pbio.0030402 16279838 PMC1283536

[B82] Kaplan R. Doeller C. F. Barnes G. R. Litvak V. Düzel E. Bandettini P. A.. (2012). Movement-related theta rhythm in humans: coordinating self-directed hippocampal learning. PLoS. Biol. 10:e1001267. 10.1371/journal.pbio.1001267 22389627 PMC3289589

[B83] Kelley W. M. Macrae C. N. Wyland C. L. Caglar S. Inati S. Heatherton T. F. (2002). Finding the self? An event-related fMRI study. J. Cogn. Neurosci. 14, 785–794. 10.1162/08989290260138672 12167262

[B84] Kelsey J. E. Vargas H. (1993). Medial septal lesions disrupt spatial, but not nonspatial, working memory in rats. Behav. Neurosci. 107, 565–574. 10.1037/0735-7044.107.4.565 8397861

[B85] Kim H. (2012). A dual-subsystem model of the brain's default network: self-referential processing, memory retrieval processes, and autobiographical memory retrieval. Neuroimage 61, 966–977. 10.1016/j.neuroimage.2012.03.025 22446489

[B86] Klein S. B. (2013). Making the case that episodic recollection is attributable to operations occurring at retrieval rather than to content stored in a dedicated subsystem of long-term memory. Front. Behav. Neurosci. 7:3. 10.3389/fnbeh.2013.00003 23378832 PMC3561741

[B87] Kroes M. C. Fernández G. (2012). Dynamic neural systems enable adaptive, flexible memories. Neurosci. Biobehav. Rev. 36, 1646–1666. 10.1016/j.neubiorev.2012.02.014 22874579

[B88] Kumaran D. Maguire E. A. (2006). An unexpected sequence of events: mismatch detection in the human hippocampus. PLoS Biol. 4:e424. 10.1371/journal.pbio.0040424 17132050 PMC1661685

[B89] Kumaran D. Maguire E. A. (2007a). Which computational mechanisms operate in the hippocampus during novelty detection? Hippocampus 17, 735–748. 10.1002/hipo.20326 17598148

[B90] Kumaran D. Maguire E. A. (2007b). Match–mismatch processes underlie human hippocampal responses to associative novelty. J. Neurosci. 27, 8517–8524. 10.1523/JNEUROSCI.1677-07.2007 17687029 PMC2572808

[B91] Kumaran D. Maguire E. A. (2009). Novelty signals: a window into hippocampal information processing. Trends Cogn. Sci. 13, 47–54. 10.1016/j.tics.2008.11.004 19135404

[B92] Laroche S. Davis S. Jay T. M. (2000). Plasticity at hippocampal to prefrontal cortex synapses: dual roles in working memory and consolidation. Hippocampus 10, 438–446. 10.1002/1098-1063(2000)10:4<438::AID-HIPO10>3.0.CO;2-3 10985283

[B93] Lau H. C. Rogers R. D. Haggard P. Passingham R. E. (2004). Attention to intention. Science 303, 1208–1210. 10.1126/science.1090973 14976320

[B94] Ledoux A. A. Phillips J. L. Labelle A. Smith A. Bohbot V. D. Boyer P. (2013). Decreased fMRI activity in the hippocampus of patients with schizophrenia compared to healthy control participants, tested on a wayfinding task in a virtual town. Psychiatr. Res. 211, 47–56. 10.1016/j.pscychresns.2012.10.005 23352276

[B95] Lever C. Burton S. Jeewajee A. Wills T. J. Cacucci F. Burgess N. (2010). Environmental novelty elicits a later theta phase of firing in CA1 but not subiculum. Hippocampus 20, 229–234. 10.1002/hipo.20671 19623610 PMC3173854

[B96] Lewis P. R. Shute C. C. D. (1967). The cholinergic limbic system: projections to hippocampal formation, medial cortex, nuclei of the ascending cholinergic reticular system, and the subfornical organ and supra-optic crest. Brain 90, 521–540. 10.1093/brain/90.3.521 6058141

[B97] Lisman J. E. Grace A. A. (2005). The hippocampal-VTA loop: Controlling the entry of information into long-term memory. Neuron 46, 703–713. 10.1016/j.neuron.2005.05.002 15924857

[B98] Long L. L. Bunce J. G. Chrobak J. J. (2015). Theta variation and spatiotemporal scaling along the septotemporal axis of the hippocampus. Front. Syst. Neurosci. 9:37. 10.3389/fnsys.2015.00037 25852496 PMC4360780

[B99] Manns J. R. Eichenbaum H. (2006). Evolution of declarative memory. Hippocampus 16, 795–808. 10.1002/hipo.20205 16881079

[B100] Markus E. J. Qin Y. L. Leonard B. Skaggs W. E. McNaughton B. L. Barnes C. A. (1995). Interactions between location and task affect the spatial and directional firing of hippocampal neurons. J. Neurosci. 15, 7079–7094. 7472463 10.1523/JNEUROSCI.15-11-07079.1995 PMC6578055

[B101] Martin-Ordas G. Call J. (2013). Episodic memory: a comparative approach. Front. Behav. Neurosci. 7:63. 10.3389/fnbeh.2013.00063 23781179 PMC3678104

[B102] McDonald R. J. White N. M. (1995). Hippocampal and nonhippocampal contributions to place learning in rats. Behav. Neurosci. 109, 579–593. 10.1037/0735-7044.109.4.579 7576202

[B103] McNaughton B. L. Barnes C. A. Gerrard J. L. Gothard K. Jung M. W. Knierim J. J.. (1996). Deciphering the hippocampal polyglot: the hippocampus as a path integration system. J. Exp. Biol. 199, 173–185. 8576689 10.1242/jeb.199.1.173

[B104] McNaughton N. (2006). The role of the subiculum within the behavioural inhibition system. Behav. Brain Res. 174, 232–250. 10.1016/j.bbr.2006.05.037 16887202

[B105] M'Harzi M. Jarrard L. E. (1992). Effects of medial and lateral septal lesions on acquisition of a place and cue radial maze task. Behav. Brain Res. 49, 159–165. 10.1016/S0166-4328(05)80160-3 1388809

[B106] Miller A. M. Vedder L. C. Law L. M. Smith D. M. (2014). Cues, context, and long-term memory: the role of the retrosplenial cortex in spatial cognition. Front. Hum. Neurosci. 8:586. 10.3389/fnhum.2014.00586 25140141 PMC4122222

[B107] Miller E. K. Cohen J. D. (2001). An integrative theory of prefrontal cortex function. Ann. Rev. Neurosci. 24, 167–202 10.1146/annurev.neuro.24.1.167 11283309

[B108] Miller G. A. Galanter E. Pribram K. H. (1960). Plans and the Structure of Behavior. New York, NY: Holt, Rinehart, & Winston.

[B109] Mishkin M. Suzuki W. A. Gadian D. G. Vargha-Khadem F. (1997). Hierarchical organization of cognitive memory. Philos. Trans. R. Soc. B 352, 1461–1467. 10.1098/rstb.1997.0132 9368934 PMC1692056

[B110] Miyachi S. Lu X. Inoue S. Iwasaki T. Koike S. Nambu A.. (2005). Organization of multisynaptic inputs from prefrontal cortex to primary motor cortex as revealed by retrograde transneuronal transport of rabies virus. J. Neurosci. 25, 2547–2556. 10.1523/JNEUROSCI.4186-04.2005 15758164 PMC6725170

[B111] Mizumori S. J. Jo Y. S. (2013). Homeostatic regulation of memory systems and adaptive decisions. Hippocampus 23, 1103–1124. 10.1002/hipo.22176 23929788 PMC4165303

[B112] Morris R. Pandya D. N. Petrides M. (1999). Fiber system linking the mid-dorsolateral frontal cortex with the retrosplenial/presubicular region in the rhesus monkey. J. Compar. Neurol. 407, 183–192. 10213090 10.1002/(sici)1096-9861(19990503)407:2<183::aid-cne3>3.0.co;2-n

[B113] Moser M. B. Moser E. I. (1998). Functional differentiation in the hippocampus. Hippocampus 8, 608–619. 9882018 10.1002/(SICI)1098-1063(1998)8:6<608::AID-HIPO3>3.0.CO;2-7

[B114] Muller R. U. Kubie J. L. (1989). The firing of hippocampal place cells predicts the future position of freely moving rats. J. Neurosci. 9, 4101–4110. 2592993 10.1523/JNEUROSCI.09-12-04101.1989 PMC6569642

[B115] Numan R. (1972). The Effects of Frontal and Septal Ablation on Response Regulation in the Cat (Doctoral dissertation, University of Tennessee, 1972). *Dissertation Abstracts International*, 1973, 33, (no. 11), 5545B–5546B. (University Microfilms no. 73–12. 426). Full text available online at: http://trace.tennessee.edu/utk_graddis/2993

[B116] Numan R. (1978). Cortical-limbic mechanisms and response control: a theoretical review. Physiol. Psychol. 6, 445–470. 10.3758/BF03326750

[B117] Numan R. (1991). Medial septal lesions impair performance on a preoperatively acquired delayed alternation task. Brain Res. Bull. 26, 449–453. 10.1016/0361-9230(91)90023-D 2049614

[B118] Numan R. (2000). Septal modulation of the working memory for voluntary behavior, in The Behavioral Neuroscience of the Septal Region, ed Numan R. (New York, NY: Springer-Verlag), 298–326.

[B119] Numan R. Feloney M. P. Pham K. H. Tieber L. M. (1995). Effects of medial septal lesions on an operant go/no-go delayed response alternation task in rats. Physiol. Behav. 58, 1263–1271. 10.1016/0031-9384(95)02044-6 8623030

[B120] Numan R. Klis D. (1992). Effects of medial septal lesions on an operant delayed go/no-go discrimination in rats. Brain Res. Bull. 29, 643–650. 10.1016/0361-9230(92)90133-I 1422861

[B121] Numan R. Lubar J. F. (1974). Role of the proreal gyrus and septal area in response modulation in the cat. Neuropsychologia 12, 219–234. 10.1016/0028-3932(74)90007-4 4601845

[B122] Numan R. Ouimette A. S. Holloway K. A. Curry C. E. (2004). Effects of medial septal lesions on action-outcome associations in rats under conditions of delayed reinforcement. Behav. Neurosci. 118, 1240–1252. 10.1037/0735-7044.118.6.1240 15598133

[B123] Numan R. Quaranta J. R. (1990). Effects of medial septal lesions on operant delayed alternation in rats. Brain Res. 531, 232–241. 10.1016/0006-8993(90)90779-B 2289124

[B124] O'Keefe J. Dostrovsky J. (1971). The hippocampus as a spatial map. Preliminary evidence from unit activity in the freely-moving rat. Brain Res. 34, 171–175. 10.1016/0006-8993(71)90358-1 5124915

[B125] O'Keefe J. Nadel L. (1978). The Hippocampus As a Cognitive Map. Oxford: Oxford University Press.

[B126] Olton D. S. Walker J. A. Wolf W. A. (1982). A disconnection analysis of hippocampal function. Brain Res. 233, 241–253. 10.1016/0006-8993(82)91200-8 7059809

[B127] Olvera-Cortés E. Cervantes M. Gonzalez-Burgos I. (2002). Place-learning, but not cue-learning training, modifies the hippocampal theta rhythm in rats. Brain Res. Bull. 58, 261–270. 10.1016/S0361-9230(02)00769-4 12128151

[B128] O'Neill P. K. Gordon J. A. Sigurdsson T. (2013). Theta oscillations in the medial prefrontal cortex are modulated by spatial working memory and synchronize with the hippocampus through its ventral subregion. J. Neurosci. 33, 14211–14224. 10.1523/JNEUROSCI.2378-13.2013 23986255 PMC3756763

[B129] Packard M. G. Hirsh R. White N. M. (1989). Differential effects of fornix and caudate nucleus lesions on two radial maze tasks: evidence for multiple memory systems. J. Neurosci. 9, 1465–1472. 2723738 10.1523/JNEUROSCI.09-05-01465.1989 PMC6569845

[B130] Pang K. C. Jiao X. Sinha S. Beck K. D. Servatius R. J. (2011). Damage of GABAergic neurons in the medial septum impairs spatial working memory and extinction of active avoidance: effects on proactive interference. Hippocampus 21, 835–846. 10.1002/hipo.20799 20865731 PMC3010529

[B131] Paus T. (2001). Primate anterior cingulate cortex: where motor control, drive and cognition interface. Nat. Rev. Neurosci. 2, 417–424. 10.1038/35077500 11389475

[B132] Penley S. C. Hinman J. R. Long L. L. Markus E. J. Escabí M. A. Chrobak J. J. (2013). Novel space alters theta and gamma synchrony across the longitudinal axis of the hippocampus. Front. Syst. Neurosci. 7:20. 10.3389/fnsys.2013.00020 23805081 PMC3691506

[B133] Philippi C. L. Duff M. C. Denburg N. L. Tranel D. Rudrauf D. (2012). Medial PFC damage abolishes the self-reference effect. J. Cogn. Neurosci. 24, 475–481. 10.1162/jocn_a_00138 21942762 PMC3297026

[B134] Poulet J. F. Hedwig B. (2007). New insights into corollary discharges mediated by identified neural pathways. Trends Neurosci. 30, 14–21. 10.1016/j.tins.2006.11.005 17137642

[B135] Prasad J. A. Chudasama Y. (2013). Viral tracing identifies parallel disynaptic pathways to the hippocampus. J. Neurosci. 33, 8494–8503. 10.1523/JNEUROSCI.5072-12.2013 23658186 PMC6619633

[B136] Ranganath C. Minzenberg M. J. Ragland J. D. (2008). The cognitive neuroscience of memory function and dysfunction in schizophrenia. Biol. Psychiatry 64, 18–25. 10.1016/j.biopsych.2008.04.011 18495087 PMC2474810

[B137] Roland J. J. Stewart A. L. Janke K. L. Gielow M. R. Kostek J. A. Savage L. M.. (2014). Medial septum-diagonal band of Broca (MSDB) GABAergic regulation of hippocampal acetylcholine efflux is dependent on cognitive demands. J. Neurosci. 34, 506–514. 10.1523/JNEUROSCI.2352-13.2014 24403150 PMC3870934

[B138] Rondi-Reig L. Petit G. H. Tobin C. Tonegawa S. Mariani J. Berthoz A. (2006). Impaired sequential egocentric and allocentric memories in forebrain-specific–NMDA receptor knock-out mice during a new task dissociating strategies of navigation. J. Neurosci. 26, 4071–4081. 10.1523/JNEUROSCI.3408-05.2006 16611824 PMC6673881

[B139] Sakimoto Y. Hattori M. Takeda K. Okada K. Sakata S. (2013). Hippocampal theta wave activity during configural and non-configural tasks in rats. Exp. Brain Res. 225, 177–185. 10.1007/s00221-012-3359-2 23224700

[B140] Sauseng P. Griesmayr B. Freunberger R. Klimesch W. (2010). Control mechanisms in working memory: a possible function of EEG theta oscillations. Neurosci. Biobehav. Rev. 34, 1015–1022. 10.1016/j.neubiorev.2009.12.006 20006645

[B141] Save E. Cressant A. Thinus-Blanc C. Poucet B. (1998). Spatial firing of hippocampal place cells in blind rats. J. Neurosci. 18, 1818–1826. 9465006 10.1523/JNEUROSCI.18-05-01818.1998 PMC6792629

[B142] Schacter D. L. Addis D. R. Hassabis D. Martin V. C. Spreng R. N. Szpunar K. K. (2012). The future of memory: remembering, imagining, and the brain. Neuron 76, 677–694. 10.1016/j.neuron.2012.11.001 23177955 PMC3815616

[B143] Schmidt B. Hinman J. R. Jacobson T. K. Szkudlarek E. Argraves M. Escabí M. A.. (2013). Dissociation between dorsal and ventral hippocampal theta oscillations during decision-making. J. Neurosci. 33, 6212–6224. 10.1523/JNEUROSCI.2915-12.2013 23554502 PMC6618918

[B144] Schultz W. (2002). Getting formal with dopamine and reward. Neuron 36, 241–263. 10.1016/S0896-6273(02)00967-4 12383780

[B145] Sharp P. E. Blair H. T. Etkin D. Tzanetos D. B. (1995). Influences of vestibular and visual motion information on the spatial firing patterns of hippocampal place cells. J. Neurosci. 15, 173–189. 7823128 10.1523/JNEUROSCI.15-01-00173.1995 PMC6578264

[B146] Siapas A. G. Lubenov E. V. Wilson M. A. (2005). Prefrontal phase locking to hippocampal theta oscillations. Neuron 46, 141–151. 10.1016/j.neuron.2005.02.028 15820700

[B147] Simons J. S. Spiers H. J. (2003). Prefrontal and medial temporal lobe interactions in long-term memory. Nat. Rev. Neurosci. 4, 637–648. 10.1038/nrn1178 12894239

[B148] Song E. Y. Kim Y. B. Kim Y. H. Jung M. W. (2005). Role of active movement in place−specific firing of hippocampal neurons. Hippocampus 15, 8–17. 10.1002/hipo.20023 15390169

[B149] Spreng R. N. Stevens W. D. Chamberlain J. P. Gilmore A. W. Schacter D. L. (2010). Default network activity, coupled with the frontoparietal control network, supports goal-directed cognition. Neuroimage 53, 303–317. 10.1016/j.neuroimage.2010.06.016 20600998 PMC2914129

[B150] Stephan K. E. Friston K. J. Frith C. D. (2009). Dysconnection in schizophrenia: from abnormal synaptic plasticity to failures of self-monitoring. Schizophr. Bull. 35, 509–527. 10.1093/schbul/sbn176 19155345 PMC2669579

[B151] Terrazas A. Krause M. Lipa P. Gothard K. M. Barnes C. A. McNaughton B. L. (2005). Self-motion and the hippocampal spatial metric. J. Neurosci. 25, 8085–8096. 10.1523/JNEUROSCI.0693-05.2005 16135766 PMC6725460

[B152] Thakkar K. N. Schall J. D. Heckers S. Park S. (2015). Disrupted saccadic corollary discharge in schizophrenia. J. Neurosci. 35, 9935–9945. 10.1523/JNEUROSCI.0473-15.2015 26156994 PMC4495243

[B153] Thierry A. M. Gioanni Y. Dégénétais E. Glowinski J. (2000). Hippocampo-prefrontal cortex pathway: anatomical and electrophysiological characteristics. Hippocampus 10, 411–419. 10.1002/1098-1063(2000)10:4<411::AID-HIPO7>3.0.CO;2-A 10985280

[B154] Tulving E. (2002). Episodic memory: from mind to brain. Annu. Rev. Psychol. 53, 1–25. 10.1146/annurev.psych.53.100901.135114 11752477

[B155] Tulving E. Markowitsch H. J. (1998). Episodic and declarative memory: role of the hippocampus. Hippocampus 8, 198–204. 9662134 10.1002/(SICI)1098-1063(1998)8:3<198::AID-HIPO2>3.0.CO;2-G

[B156] van der Meer M. Kurth-Nelson Z. Redish A. D. (2012). Information processing in decision-making systems. Neuroscientist 18, 342–359. 10.1177/1073858411435128 22492194 PMC4428660

[B157] Vanderwolf C. H. (1969). Hippocampal electrical activity and voluntary movement in the rat. Electroencephalogr. Clin. Neurophysiol. 26, 407–418. 10.1016/0013-4694(69)90092-3 4183562

[B158] Vanderwolf C. H. (1971). Limbic-diencephalic mechanisms of voluntary movement. Psychol. Rev. 78, 83–113. 10.1037/h0030672 5547375

[B159] Van Hoesen G. W. Pandya D. N. Butters N. (1972). Cortical afferents to the entorhinal cortex of the rhesus monkey. Science 175, 1471–1473. 10.1126/science.175.4029.1471 4622430

[B160] Vann S. D. Aggleton J. P. Maguire E. A. (2009). What does the retrosplenial cortex do? Nat. Rev. Neurosci. 10, 792–802. 10.1038/nrn2733 19812579

[B161] Vertes R. P. (2006). Interactions among the medial prefrontal cortex, hippocampus and midline thalamus in emotional and cognitive processing in the rat. Neuroscience 142, 1–20. 10.1016/j.neuroscience.2006.06.027 16887277

[B162] Veyrac A. Allerborn M. Gros A. Michon F. Raguet L. Kenney J.. (2015). Memory of occasional events in rats: individual episodic memory profiles, flexibility, and neural substrate. J. Neurosci. 35, 7575–7586. 10.1523/JNEUROSCI.3941-14.2015 25972182 PMC6705429

[B163] Vinogradova O. S. (1970). Registration of information and the limbic system, in Short-term Changes in Neural Activity and Behavior, eds Horn G. Hinde R. A. (Cambridge: Cambridge University Press), 95–140.

[B164] Vinogradova O. S. (1975). Functional organization of the limbic system in the process of registration of information: facts and hypotheses, in The Hippocampus, Vol. 2, eds Isaacson R. L. Pribram K. H. (New York, NY: Plenum Press), 3–69.

[B165] Vinogradova O. S. (2001). Hippocampus as comparator: role of the two input and two output systems of the hippocampus in selection and registration of information. Hippocampus 11, 578–598. 10.1002/hipo.1073 11732710

[B166] Von Holst E. (1954). Relations between the central nervous system and the peripheral organs. Br. J. Anim. Behav. 2, 89–94. 10.1016/S0950-5601(54)80044-X

[B167] Voss J. L. Gonsalves B. D. Federmeier K. D. Tranel D. Cohen N. J. (2011). Hippocampal brain-network coordination during volitional exploratory behavior enhances learning. Nat. Neurosci. 14, 115–120. 10.1038/nn.2693 21102449 PMC3057495

[B168] Walsh T. J. (2000). The medial septum and working/episodic memory, in The Behavioral Neuroscience of the Septal Region, ed Numan R. (New York, NY: Springer-Verlag), 327–362.

[B169] Wheeler M. A. Stuss D. T. Tulving E. (1997). Toward a theory of episodic memory: the frontal lobes and autonoetic consciousness. Psychol. Bull. 121, 331–354. 10.1037/0033-2909.121.3.331 9136640

[B170] Whishaw I. Q. (2000). The septohippocampal system and path integration, in The Behavioral Neuroscience of the Septal Region, ed Numan R. (New York, NY: Springer-Verlag), 270–297.

[B171] Whishaw I. Q. Wallace D. G. (2003). On the origins of autobiographical memory. Behav. Brain Res. 138, 113–119. 10.1016/S0166-4328(02)00236-X 12527442

[B172] Whitford T. J. Ford J. M. Mathalon D. H. Kubicki M. Shenton M. E. (2012). Schizophrenia, myelination, and delayed corollary discharges: a hypothesis. Schizophr. Bull. 38, 486–494. 10.1093/schbul/sbq105 20855415 PMC3329979

[B173] Wiener S. I. Korshunov V. A. (1995). Place-independent behavioural correlates of hippocampal neurones in rats. Neuroreport 7, 183–188. 10.1097/00001756-199512000-00044 8742447

[B174] Wolf D. H. Gur R. C. Valdez J. N. Loughead J. Elliott M. A. Gur R. E.. (2007). Alterations of fronto-temporal connectivity during word encoding in schizophrenia. Psychiatr. Res. 154, 221–232. 10.1016/j.pscychresns.2006.11.008 17360163 PMC2359768

[B175] Wood E. R. Dudchenko P. A. Robitsek R. J. Eichenbaum H. (2000). Hippocampal neurons encode information about different types of memory episodes occurring in the same location. Neuron 27, 623–633. 10.1016/S0896-6273(00)00071-4 11055443

[B176] Yang S. Yang S. Moreira T. Hoffman G. Carlson G. C. Bender K. J.. (2014). Interlamellar CA1 network in the hippocampus. Proc. Natl. Acad. Sci. U.S.A. 111, 12919–12924. 10.1073/pnas.1405468111 25139992 PMC4156755

[B177] Young B. McNaughton N. (2000). Common firing patterns of hippocampal cells in a differential reinforcement of low rates of response schedule. J. Neurosci. 20, 7043–7051. 10995850 10.1523/JNEUROSCI.20-18-07043.2000 PMC6772825

[B178] Zola S. M. Mahut H. (1973). Paradoxical facilitation of object reversal learning after transection of the fornix in monkeys. Neuropsychologia 11, 271–284. 10.1016/0028-3932(73)90038-9 4792178

[B179] Zola-Morgan S. Squire L. R. (1993). Neuroanatomy of memory. Ann. Rev. Neurosci. 16, 547–563. 10.1146/annurev.ne.16.030193.002555 8460903

[B180] Zou D. Aitake M. Hori E. Umeno K. Fukuda M. Ono T.. (2009). Rat hippocampal theta rhythm during sensory mismatch. Hippocampus 19, 350–359. 10.1002/hipo.20524 18958848

